# Construction of Global Acyl Lipid Metabolic Map by Comparative Genomics and Subcellular Localization Analysis in the Red Alga *Cyanidioschyzon merolae*

**DOI:** 10.3389/fpls.2016.00958

**Published:** 2016-06-30

**Authors:** Natsumi Mori, Takashi Moriyama, Masakazu Toyoshima, Naoki Sato

**Affiliations:** ^1^Department of Life Sciences, Graduate School of Arts and Sciences, University of TokyoTokyo, Japan; ^2^Japan Science and Technology Agency, Core Research for Evolutional Science and TechnologyTokyo, Japan

**Keywords:** comparative genomics, subcellular localization, lipid metabolism, red alga, *Cyanidioschyzon merolae*

## Abstract

Pathways of lipid metabolism have been established in land plants, such as *Arabidopsis thaliana*, but the information on exact pathways is still under study in microalgae. In contrast with *Chlamydomonas reinhardtii*, which is currently studied extensively, the pathway information in red algae is still in the state in which enzymes and pathways are estimated by analogy with the knowledge in plants. Here we attempt to construct the entire acyl lipid metabolic pathways in a model red alga, *Cyanidioschyzon merolae*, as an initial basis for future genetic and biochemical studies, by exploiting comparative genomics and localization analysis. First, the data of whole genome clustering by Gclust were used to identify 121 acyl lipid-related enzymes. Then, the localization of 113 of these enzymes was analyzed by GFP-based techniques. We found that most of the predictions on the subcellular localization by existing tools gave erroneous results, probably because these tools had been tuned for plants or green algae. The experimental data in the present study as well as the data reported before in our laboratory will constitute a good training set for tuning these tools. The lipid metabolic map thus constructed show that the lipid metabolic pathways in the red alga are essentially similar to those in *A. thaliana*, except that the number of enzymes catalyzing individual reactions is quite limited. The absence of fatty acid desaturation to produce oleic and linoleic acids within the plastid, however, highlights the central importance of desaturation and acyl editing in the endoplasmic reticulum, for the synthesis of plastid lipids as well as other cellular lipids. Additionally, some notable characteristics of lipid metabolism in *C. merolae* were found. For example, phosphatidylcholine is synthesized by the methylation of phosphatidylethanolamine as in yeasts. It is possible that a single 3-ketoacyl-acyl carrier protein synthase is involved in the condensation reactions of fatty acid synthesis in the plastid. We will also discuss on the redundant β-oxidation enzymes, which are characteristic to red algae.

## Introduction

Oil production by microalgae is a recent trend, but very little is known about the lipid metabolic pathways in microalgae, except in the green alga *Chlamydomonas reinhardtii*, in which considerable knowledge is rapidly accumulating (Li-Beisson et al., 2015). The researchers on microalgae still use obscure views on the lipid metabolic pathways, which are mostly the products of analogy with the pathways obtained in better-studied organisms, such as *Arabidopsis thaliana* and *C. reinhardtii*. The overview on the lipid metabolism in plants (mostly *Arabidopsis*) is summarized below as a reference (Supplementary Figure 1).

Plastids are the major site of fatty acid synthesis. 16- or 18-carbon fatty acids, such as palmitic, stearic and oleic acids, are synthesized in the plastids (Joyard et al., 2010). Mitochondrial fatty acid synthesis is also known as a minor pathway (Wada et al., 1997; Gueguen et al., 2000). Very-long-chain fatty acids (more than 20-carbon fatty acids) are synthesized in the endoplasmic reticulum (ER) by elongation of 16- or 18-carbon acyl-coenzyme A (CoA) produced in the plastids (Haslam and Kunst, 2013). Four major plastid membrane lipids, monogalactosyldiacylglycerol (MGDG), digalactosyldiacylglycerol (DGDG), sulfoquinovosyldiacylglycerol (SQDG) and phosphatidylglycerol (PG), are synthesized within the plastids (Joyard et al., 2010). On the other hand, the ER is the major site of synthesis of phospholipids, namely, phosphatidylcholine (PC), phosphatidylethanolamine (PE), phosphatidylserine (PS), phosphatidylinositol (PI) and PG, as well as the site of triacylglycerol (TAG) synthesis in plants (Li-Beisson et al., 2013). The glycolipids and phospholipids are further modified by formation of double bonds within their acyl chains, catalyzed by various acyl lipid desaturases in the plastids and the ER. Once synthesized, TAG is accumulated in the organelle called lipid body and is used as energy and carbon sources during germination (Chapman et al., 2012). Peroxisomes are the major site of β-oxidation pathway in plants (Graham and Eastmond, 2002; Poirier et al., 2006).

We are interested in red algae as new resources for future bioengineering. Red algae have attracted less attention in biochemical and genomic researches, mainly because many of them are marine macrophytes (Nakamura et al., 2013). In contrast, *Cyanidioschyzon merolae* is a unicellular red alga, which is easy to grow and to manipulate in the laboratory. It was originally isolated from an acidic hot spring, and grows by binary fission. Its habitat is warm (up to 50°C) and acidic (pH 1.5–2.5) land water containing sulfuric acid. This alga is a versatile model alga, because its complete sequences of the nuclear, plastid and mitochondrial genomes have been determined (Matsuzaki et al., 2004; Nozaki et al., 2007). The small number of protein coding genes (4775 nuclear genes) in *C. merolae* as well as the experimental techniques of transformation (Ohnuma et al., 2008, 2014) made it an ideal model alga for reverse-genetic analysis. In addition, its simple cellular organization (a single plastid, a single mitochondrion, a single peroxisome) makes it a suitable organism for the study of cytological analysis, such as subcellular localization of proteins.

We already published two lines of studies on *C. merolae*. In one of them lipid metabolism was analyzed by radiolabeling and a preliminary genomic analysis (Sato and Moriyama, 2007), showing various points of uniqueness in lipid metabolism has been noted. First, this alga does not possess stearoyl-acyl-carrier-protein (ACP) desaturase, which is universally present in green plants and algae. This makes ER, rather than the plastid, the major site of desaturation of fatty acids and lipids. The “coupled pathway” was proposed for the synthesis of galactolipids in the plastid. Second, *C. merolae* lacks polyunsaturated fatty acids containing three or more double bonds, such as linolenic acid and arachidonic acid. Third, this alga has MgdA and DgdA homologs involved in the galactolipid synthesis in cyanobacteria, as well as a eukaryotic type MGD1 (for a review, see Sato and Awai, 2016). A recently published work in this line (Toyoshima et al., 2016) analyzed TAG accumulation in the nitrogen deficiency. Sato et al. (2016b) studied biosynthesis of PC by labeling with radioactive phosphate.

In another line of study (Moriyama et al., 2014a), we used comparative genomics to identify all 75 enzymes involved in the central carbohydrate metabolism in *C. merolae*, which were then subjected to global, experimental analysis of subcellular localization. Although, most of these enzymes are soluble proteins, we noticed some errors in prediction of plastid or mitochondrial localization by the available prediction tools. By extending the previous study (Sato and Moriyama, 2007; Sato et al., 2016b), we now attempt to identify all lipid metabolic enzymes in *C. merolae* by comparative genomics, and to analyze experimentally the subcellular localization of all these enzymes. This is a new challenge, because a large part of these enzymes are insoluble, membrane proteins. In fact, preliminary prediction results suggested a strange compartmentation of lipid metabolic enzymes, very different from that in plants and algae. Subcellular localization of enzymes has been predicted by the computer programs, such as TargetP (Emanuelsson et al., 2000) and WoLF PSORT (Horton et al., 2007), based on neural network algorithm that gives good estimates after proper training. In this respect, the existing programs might be suitable for analysis of proteins of land plants, but not for algal proteins. Recently, the PredAlgo was made as a prediction tool of green algal proteins using datasets of proteome analysis in *C. reinhardtii* (Tardif et al., 2012). This is still not suitable for red algal proteins, as we will see later.

In the present study, we first report on the construction of a list (or database) of all enzymes involved in acyl lipid metabolism in *C. merolae* by exploiting the whole genome clustering by the Gclust software (Sato, 2009) with various photosynthetic and non-photosynthetic organisms. We identified the lipid metabolic enzymes in algae and cyanobacteria by using the orthologs in *A. thaliana* as queries. Second, we determined the subcellular localization of these enzymes by the GFP- or hemagglutinin (HA) tag-fusion techniques. Third, we created probable pathway maps of lipid metabolism based on these results. We confirmed that the major sites of lipid metabolism are plastid and ER in *C. merolae* as in plants, although the pathways in *C. merolae* consist of a minimal set of enzymes. An exception is the β-oxidation enzymes, of which we found two sets.

## Materials and methods

### Construction of comparative database of acyl lipid metabolic enzymes in algae and cyanobacteria

To build the database of lipid metabolic enzymes in algae and cyanobacteria, we searched for enzymes related to synthesis of fatty acids and lipids, lipid degradation, β-oxidation and lipid trafficking in *C. merolae* 10D, *C. reinhardtii* CC-503, *Synechocystis* sp. PCC 6803 and *Anabaena* sp. PCC 7120 using the Gclust database (Sato, 2009; http://gclust.c.u-tokyo.ac.jp/) based on the genomic data of *A. thaliana*. We used the TAIR database (http://www.arabidopsis.org/), the KEGG database (http://www.genome.jp/kegg/), *C. reinhardtii* v5.5 in the Phytozome database (http://phytozome.jgi.doe.gov) and the *Cyanidioschyzon merolae* Genome Project (http://merolae.biol.s.u-tokyo.ac.jp/) to obtain the genomic data of these organisms.

### Growth of organism

*C. merolae* cells were cultured in 100 ml of 2 × Allen's medium (pH 2.5; 20 mM (NH_4_)_2_SO_4_, 4 mM KH_2_PO_4_, 2 mM MgSO_4_, 1 mM CaCl_2_, 50 μM FeCl_3_, 10 μM ethylenediaminetetraacetic acid, 200 nM CuSO_4_, 200 nM ZnSO_4_, 160 nM Na_2_MoO_4_, 300 nM CoCl_2_, 16 μM H_3_BO_3_, 3.2 μM MnCl_2_) using shaking flasks under continuous red light (30 μmol m^−2^ s^−1^) at 40°C.

### Plasmid construction

pCG1 vector contains *APCC* promoter of *C. merolae, sGFP (S65T)* gene and *NOS* terminator (Watanabe et al., 2011). First, we replaced *sGFP (S65T)* gene with *EGFP* gene in pCG1 vector and named it pCEG1 vector. To obtain pCG1 vector without *sGFP (S65T)* gene, we performed PCR using pCG1 vector as a template and a primer set (5′-AGC GGCCGCCCGGCTGCAGATCGTTCAAACATT TG-3′ and 5′-GGA TCCTCTAGAGGTCAACGAACGAAGAAACAC AG-3′). *EGFP* gene was amplified using pEGFP vector (Clontech Laboratories, Mountain View, CA, USA) as a template and a primer set (5′-ACC TCTAGAGGATCCatggtgagcaagggcga gga-3′ and 5′ -AGC CGGGCGGCCGCTttacttgtacagctcgtc ca-3′; The sequences in uppercase letters indicate common sequences of pCEG1 vector required for the cloning using the In-Fusion Cloning Kit; Clontech Laboratories.) and, inserted downstream of *APCC* promoter in pCG1 vector without *sGFP (S65T)* gene using the In-Fusion Cloning Kit.

We prepared a sequence alignment for each lipid metabolic enzyme by Clustal X 2.0.10 (Larkin et al., 2007) and determined the region of *N*-terminal extended sequence, which is a putative transit peptide in *C. merolae*. A DNA fragment of *N*-terminal extended sequence was amplified using the *C. merolae* genome, and inserted into pCEG1 vector restricted with *Xba*I using the In-Fusion Cloning Kit. For small proteins, such as ACBP or mtACP, the GFP-fused construct was prepared using the full-length gene as an insert. For constructs of HA-tagged proteins, full length DNA fragments were inserted into pBSHAb-T3′ vector (Ohnuma et al., 2008) containing *APCC* promoter (Moriyama et al., 2014a) restricted with *Pac*I using the In-Fusion Cloning Kit. For constructs of EGFP-fused *C*-terminal peptide of β-oxidation enzymes, first we performed PCR using pCEG1 vector as a template and a primer set (5′-TAAAGCGGCCGCCCG GCTGCAGATCGTTCA-3′ and 5′-CTTGTACAGCTCGTC CATGCCGAGAGTGAT-3′). A DNA fragment of *C*-terminal peptide of β-oxidation enzymes was amplified using the *C. merolae* genome. These PCR products were cloned with the In-Fusion Cloning Kit. Supplementary Table 1 is a list of primers used for amplification of inserts.

### Transformation and immunostaining

Transformation of *C. merolae* cells was performed using the polyethylene glycol-method as described earlier (Moriyama et al., 2014b). If necessary, transformed cells expressing a GFP-fused protein were immunostained according to Moriyama et al. (2014a). *C. merolae* cells bearing HA-tagged proteins were immunostained with the Tyramide Staining Amplification (TSA) Kit (Invitrogen) according to Moriyama et al. (2014a). Transformed or immunostained cells were observed with a fluorescence microscope (Moriyama et al., 2014a). Genetic manipulation experiments were carried out according to the guideline of the Genetic Recombination Experiment Safety Control Committee in Tokyo Univerity.

## Results

### Search for lipid metabolic enzymes in *C. merolae*

We searched for enzymes related to acyl lipid metabolism in *C. merolae, C. reinhardtii, Synechocystis* sp. PCC 6803 and *Anabaena* sp. PCC 7120 using the Gclust database (Sato, 2009) using the 373 known enzymes involved in the acyl lipid metabolism in *A. thaliana* as queries (Supplementary Table 2). The Gclust software is a tool of whole genome clustering, which is suited for identifying highly related orthologs (conserving their entire domain structure) over prokaryotes and eukaryotes, allowing the presence or absence of a transit peptide. Proteins sharing a single domain might not be identified in the same cluster, but can be picked up as a related group. As a result, 121, 163, 51, and 59 putative enzymes involved in the acyl lipid metabolism were detected in *C. merolae, C. reinhardtii, Synechocystis* sp. PCC 6803 and *Anabaena* sp. PCC 7120, respectively. Table 1 is a summary of enzymes involved in the synthesis of fatty acids and lipids as well as β-oxidation in *C. merolae*. Other enzymes, such as lipases and lipid transporters in the plastid, are summarized in Supplementary Table 3.

**Table 1:** **List of acyl lipid metabolic enzymes in *C. merolae* with emphasis on the subcellular localization**.

**1. Enzyme name**	**2. Abbreviation of enzyme name and/or gene name**	**3. Locus tag**	**4. Subcellular localization**	**5. Result of prediction of subcellular localization**		
				**TargetP**	**WoLF PSORT**	**PredAlgo**
**FATTY ACID SYNTHESIS AND ELONGATION**						
Acetyl-CoA carboxylase (multifunctional type)	ACCase (ACC1)	CMM188C	Cyt	Other	Nuc, Cyt	Other
Acetyl-CoA carboxylase (multisubunit type)	ACCase (AccA)	CMV056C	Pt-genome			
	(AccB)	CMV134C	Pt-genome			
	(AccC)	CMS299C	Pt	Pt	Pt, Cyt	Mt
	(AccD)	CMV207C	Pt-genome			
Malonyl-CoA:ACP malonyltransferase	MCMT	CMT420C	Pt	Other	Pt	Pt
Acetyl-CoA:ACP acetyltransferase	ACAT	Not detected				
Acyl carrier protein	ACP (AcpP)	CMV132C	Pt-genome			
	(mtACP)	CMS372C	Mt	Mt	Pt	Mt
3-Ketoacyl-ACP synthase	KAS (KAS I)	CMM286C	Pt	Pt	Mt	Other
	(mtKAS)	CML329C	Mt	Other	Pt	Other
3-Ketoacyl-ACP reductase	KAR	CMS393C	Pt	Pt	Pt	Other
3-Hydroxyacyl-ACP dehydratase	HAD	CMI240C	Pt	Mt	Pt	Other
Enoyl-ACP reductase	EAR	CMT381C	Pt	Mt	Pt	Pt
Acyl-ACP thioesterase	AAT	CMH111C	Pt	Pt	Cyt	Other
3-Ketoacyl-CoA synthase (Elongase)	KCS	CMD118C	ER	Pt	PM	Mt
3-Ketoacyl-CoA reductase	KCR	CMK172C	ER	Other	Pt	SP
3-Hydroxyacyl-CoA dehydratase	HCD	CMR006C	ER	SP	PM	Other
Enoyl-CoA reductase	ECR	CMD146C	ER	Mt	Pt	Mt
Homologs of yeast ELO genes (3-Ketoacyl-CoA synthase?)	ELO-like	CMT175C	ER	Mt	Pt	Other
		CMM126C	ER	Other	Cyt	Other
		CML178C	Not detected	Mt	Pt	Pt
**DESATURASE**						
Stearoyl-CoA desaturase	SCD	CMM045C [1-2]	ER	Other	Cyt	Other
Acyl lipid Δ9 desaturase	Δ9Des	CMJ201C [1]	ER	Other	PM	Pt
Acyl lipid Δ12 desaturase	Δ12Des	CMK291C [1]	ER	Other	Pt, Pt_Mt	Other
Phosphatidylglycerol specific Δ3-*trans* desaturase	FAD4	CMF187C	Not detected	Other	Nuc	Other
**SYNTHESIS OF GLYCOLIPIDS AND PG**						
Glycerol-3-phosphate acyltransferase	GPAT	CMJ027C	Pt	Mt	Pt	Pt
Lysophosphatidic acid acyltransferase	LPAT	CMF185C	Pt	Mt	PM	Mt
Phosphatidic acid phosphatase	PAP	Not detected				
Monogalactosyldiacylglycerol synthase	MGD1	CMI271C	Pt	Pt	Pt	Pt
Monoglucosyldiacylglycerol synthase	MgdA	CMT267C	ER	Mt	PM	SP
Glucolipid epimerase	MgdE	Not detected				
Digalactosyldiacylglycerol synthase	DgdA	CMV121C [3]	Pt-genome			
Galactolipid:galactolipid galactosyltransferase	SFR2	Not detected				
UDP-sulfoquinovose synthase	SQD1	CMR012C [5]	Pt	Pt	Pt	Pt
Sulfoquinovosyldiacylglycerol synthase	SQD2	CMR015C [5]	Pt	Pt	Pt	Mt
CDP-diacylglycerol synthase	CDP-DAGS	CMS056C	Pt	Pt	PM	Pt
		CMM311C	Pt	Pt	PM	Pt
Phosphatidylglycerolphosphate synthase	PGPS	CMJ134C	Pt, Cyt	Other	PM	Other
Phosphatidylglycerolphosphate phosphatase	PGPP	Not detected				
**SYNTHESIS OF PHOSPHOLIPIDS AND TAG**						
Glycerol-3-phosphate acyltransferase	GPAT	CMK217C	ER	Mt	PM	Other
		CMA017C	Cyt	Other	PM	Other
Lysophosphatidic acid acyltransferase	LPAT	CMJ021C	ER	Mt	Pt	Other
		CMS008C	Cyt	Mt	PM	Mt
		CME109C	ER	Other	PM	SP
Phosphatidic acid phosphatase	PAP	CMR054C	ER	Other	PM	SP
		CMR488C	ER	Other	PM	SP
		CMT106C	Cyt	Other	Nuc	Other
		CMT239C	ER	SP	Nuc	SP
		CMN061C	ER	Other	PM	Mt
CDP-diacylglycerol synthase	CDP-DAGS	CMN215C	Cyt	Other	PM	Other
Choline kinase	CK	Not detected				
Phosphocholine cytidylyltransferase	CCT	Not detected				
CDP-choline:diacylglycerol cholinephosphotransferase	CPT	EPT [6]				
Phosphoethanolamine methyltransferase	PEAMT	Not detected				
Ethanolamine kinase	EK	CMR011C	ER	Other	Cyt	Other
Phosphoethanolamine cytidylyltransferase	ECT	CMS052C	Mt	Mt	Pt	SP
CDP-ethanolamine:diacylglycerol ethanolaminephosphotransferase	EPT	CMF133C	Ves	Other	PM	Other
Phosphatidylethanolamine methyltransferase	PEMT	CMF090C	Cyt	Pt	PM	Pt
Phospholipid methyltransferase	PLMT	CMP111C	ER	Other	Pt	SP
		CMA134C	ER	Other	PM	SP
Phosphatidylserine synthase	PSS	Not detected				
Phosphatidylserine decarboxylase	PSD	CMK243C	ER	Mt	Mt	Other
Phosphatidylinositol synthase	PIS	CMM125C	Cyt	SP	PM	Other
Cardiolipin synthase	CLS	CMN196C [4]	Cyt	SP	Pt	Other
Lysocardiolipin acyltransferase	TAZ1	CMP142C	ER, CM	Mt	Pt	Mt
Diacylglycerol acyltransferase	DGAT	CMQ199C	ER	Mt	Pt	Other
		CME100C	Cyt	Mt	PM	Mt
		CMJ162C	ER	SP	Mt	SP
Phospholipid:diacylglycerol acyltransferase	PDAT	Not detected				
Phosphatidylcholine:diacylglycerol cholinephosphotransferase	PDCT	Not detected				
Lysophospholipid acyltransferase	LPLAT	CMI139C	ER	Mt	Pt	Pt
		CMR130C	ER	Other	PM	SP
Acyltransferase?		CMB069C	ER	Pt	Pt	Pt
**β-OXIDATION**						
Acyl-CoA oxidase	ACX	CMK115C	Cyt/Cyt	Other	Cyt	Pt
Acyl-CoA dehydrogenase	ACDH	CML080C	Mt	Mt	Mt	Other
Isovaleryl-CoA dehydrogenase	IVD	CMT072C	Mt	Mt	Nuc	Other
Enoyl-CoA hydratase	ECH	CMK139C	Mt	Mt	Pt	Mt
		CMT074C	Pt	Mt	Pt	Other
3-Hydroxyacyl-CoA dehydrogenase	HACDH	CMC137C	Mt, Cyt	Other	Pt	Mt
Multifunctional protein	MFP	CMR380C	Ves/Cyt	Other	ER, ER_PM	SP
3-Ketoacyl-CoA thioesterase	KAT	CMA042C	Cyt/Ves	Other	Cyt	Mt
		CME087C	Mt	Mt	Pt	Mt

This table is a list of acyl lipid metabolic enzymes in C. merolae. Column 4 indicates the results of subcellular localization analysis in this study. Column 5 is a summary of the results of prediction of subcellular localization using three different programs. TargetP and the PredAlgo can predict plastidic, mitochondrial and secretory pathway proteins. Proteins predicted as targeted to other subcellular compartments were indicated “Other.” WoLF PSORT can predict various subcellular localizations of proteins. Results of prediction using the WoLF PSORT indicated subcellular localization(s) having the highest score. Subcellular localization of CMM311C (encoding CDP-DAGS) was not analyzed, because the amino acid sequence of this protein is identical to that of CMS056C. Subcellular localization of three β-oxidation enzymes, namely CMK115C (ACX), CMR380C (MFP), CMA042C (KAT), indicate results of analysis using constructs of GFP-fused N-terminal peptide [X] or GFP-fused C-terminal peptide [Y], such as X/Y. Abbreviations: CM, cytoplasmic membrane; Cyt, cytosol; ER, endoplasmic reticulum; Mt, mitochondrion; Nuc, nucleus; Per, peroxisome; PM, Plasma membrane; Pt, plastid; Pt-genome, genes encoded in the plastid genome; SP, secretory pathway; Ves, vesicle. References: [1], (Sato and Moriyama, 2007); [2], (Itoh et al., 1998); [3], (Sakurai et al., 2007); [4],(Katayama et al., 2004); [5], (Sato et al., 2016a) (NB: this paper is a publication from a different group from ours); [6] (Sato et al., 2016b)

We estimated subcellular localization of these enzymes using three prediction tools for subcellular localization, namely TargetP (Emanuelsson et al., 2000), WoLF PSORT (Horton et al., 2007) and PredAlgo (Tardif et al., 2012; Table 1, Supplementary Table 3). TargetP and WoLF PSORT are predictors for subcellular localization of eukaryotic proteins including plants. PredAlgo is a prediction tool for subcellular localization of green algal proteins. A number of differences were detected in the localization of *C. merolae* enzymes from that of the plant homologs. To examine if the localization of these enzymes are really different in plants and *C. merolae*, we analyzed subcellular localization of 113 putative lipid metabolic enzymes in *C. merolae* using the GFP- or HA tag-fusion techniques. Eight enzymes encoded in the plastid genome were not analyzed, because they are considered to remain in the plastid and function therein.

### Global analysis of subcellular localization of lipid metabolic enzymes in *C. merolae*

**Figure 1:**
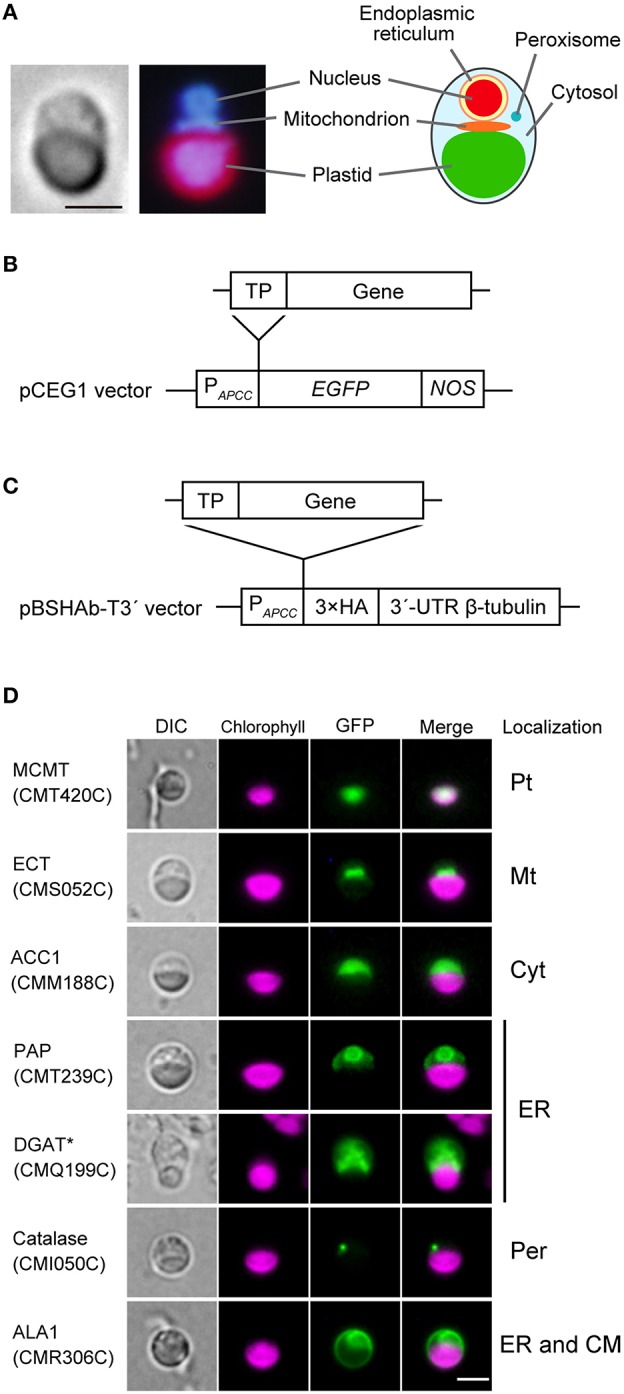
**Examples of subcellular localization of GFP-fused proteins**. **(A)** Fluorescence microscopic images showing a *C. merolae* cell stained with 4′,6-diamidino-2-phenylindole (DAPI; left). The illustration is a summary of cellular structure of *C. merolae* cell (right). Bar = 2 μm. **(B)** Structure of a construct expressing a GFP-fused protein. pCEG1 vector contains *APCC* promoter of *C. merolae* (P_*APCC*_) (Watanabe et al., 2011), gene for *enhanced green fluorescence protein* (*EGFP*) and *NOS* terminator (*NOS*). Genomic fragment of *N*-terminal transit peptide (TP) of lipid metabolic enzymes was inserted into pCEG1 vector and cloned. This construct was transformed into *C. merolae* cells for transient expression. If necessary, transformed cells were immunostained with anti-GFP antibody. **(C)** Structure of a construct expressing an HA-tagged protein. pBSHAb-T3′ vector (Ohnuma et al., 2008) contains three repeats of HA tag (3 × HA) and 3′-untranslated region (UTR) of β-tubulin of *C. merolae*. A full-length sequence of candidate genes was inserted into pBSHAb-T3′ vector containing *APCC* promoter. This construct was transformed into *C. merolae* cells, and then subcellular localization of HA-tagged proteins was detected by immunostaining using anti-HA antibody. **(D)** Fluorescence micrographs of *C. merolae* cells transiently expressing GFP-fused proteins. These images show that subcellular localization of GFP-fused proteins to plastid (Pt), mitochondrion (Mt), cytosol (Cyt), ER, peroxisome (Per), and dual localization of ER and cytoplasmic membrane (CM). GFP-fused PAP was localized in the periphery of the nucleus. In GFP-fused DGAT, GFP fluorescence was observed as a fog in cytosol. We, therefore, supposed these proteins as ER-localized enzymes. An asterisk indicates an enzyme, whose subcellular localization was examined by immunofluorescence rather than GFP fluorescence. DIC, Nomarski differential interference contrast image; Chlorophyll, autofluorescence from phycobilin and chlorophyll; GFP, GFP fluorescence or immunofluorescence using anti-GFP antibody; Merge, merged images of autofluorescence and green fluorescence from the fusion protein. Bar = 2 μm. Abbreviation of enzyme names is listed in Table 1, Supplementary Table 3.

Plastid, mitochondrion, nucleus and peroxisome are easily observed without overlapping in the *C. merolae* cell. Additionally, ER is located at the periphery of the nucleus (Yagisawa et al., 2012; Figure 1A). To determine the subcellular localization of lipid metabolic enzymes, each of the *N*-terminal transit peptide region of these enzymes was fused at the *N*-terminus of GFP (Figure 1B), or three repeats of the HA tag were fused at the *C*-terminus of full length of these enzymes (Figure 1C). The GFP- or HA tag-fused proteins were expressed under the control of *APCC* promoter of *C. merolae* (Watanabe et al., 2011). When GFP fluorescence was not detected clearly, transformed cells were fixed and immunostained with anti-GFP antibody. HA-tagged proteins were detected by immunostaining with anti-HA antibody. Figure 1D shows examples of subcellular localization of GFP-fusion proteins that were localized to the plastid (malonyl-CoA:ACP malonyltransferase; MCMT; CMT240C), the mitochondrion (phosphoethanolamine cytidylyltransferase; ECT; CMS052C), the cytosol (multifunctional type acetyl-CoA carboxylase; ACC1; CMM188C), the ER (phosphatidic acid phosphatase; PAP; CMT239C and diacylglycerol acyltransferase; DGAT; CMQ199C), the peroxisome (catalase; CMI050C; Imoto et al., 2013), and dually localized to the ER and the cytoplasmic membrane (flippase; ALA1; CMR306C). GFP-fused CMT239C was targeted to the periphery of the nucleus. In GFP-fused CMQ199C, immunofluorescence was observed as a fog in the cytosol. We supposed that both GFP-fusion proteins that were not homogeneously distributed in the cytosol were localized in the ER. In this way, the localization is unambiguously identified, because each organelle is present as a single entity. There is no complication due to crowding of organelles, which is usually the case in plant cells. Micrographs of 3-ketoacyl-CoA synthase (KCS; CMD118C, Supplementary Figure 2C), phospholipase A1 (PLA1; CMH204C, Supplementary Figure 5A), and phosphatidylinositol-4-phosphate 5-kinase (PIP5K; CME153C, Supplementary Figure 6B) were showed as examples of immunostained for ER, cytosol and both cytosol and cytoplasmic membrane in *C. merolae* cells. Other fluorescence micrographs are shown in Supplementary Figures 2–6. Results of subcellular localization analysis of lipid metabolic enzymes are summarized in Table 1, Supplementary Table 3.

### Fatty acid synthesis and elongation

**Figure 2:**
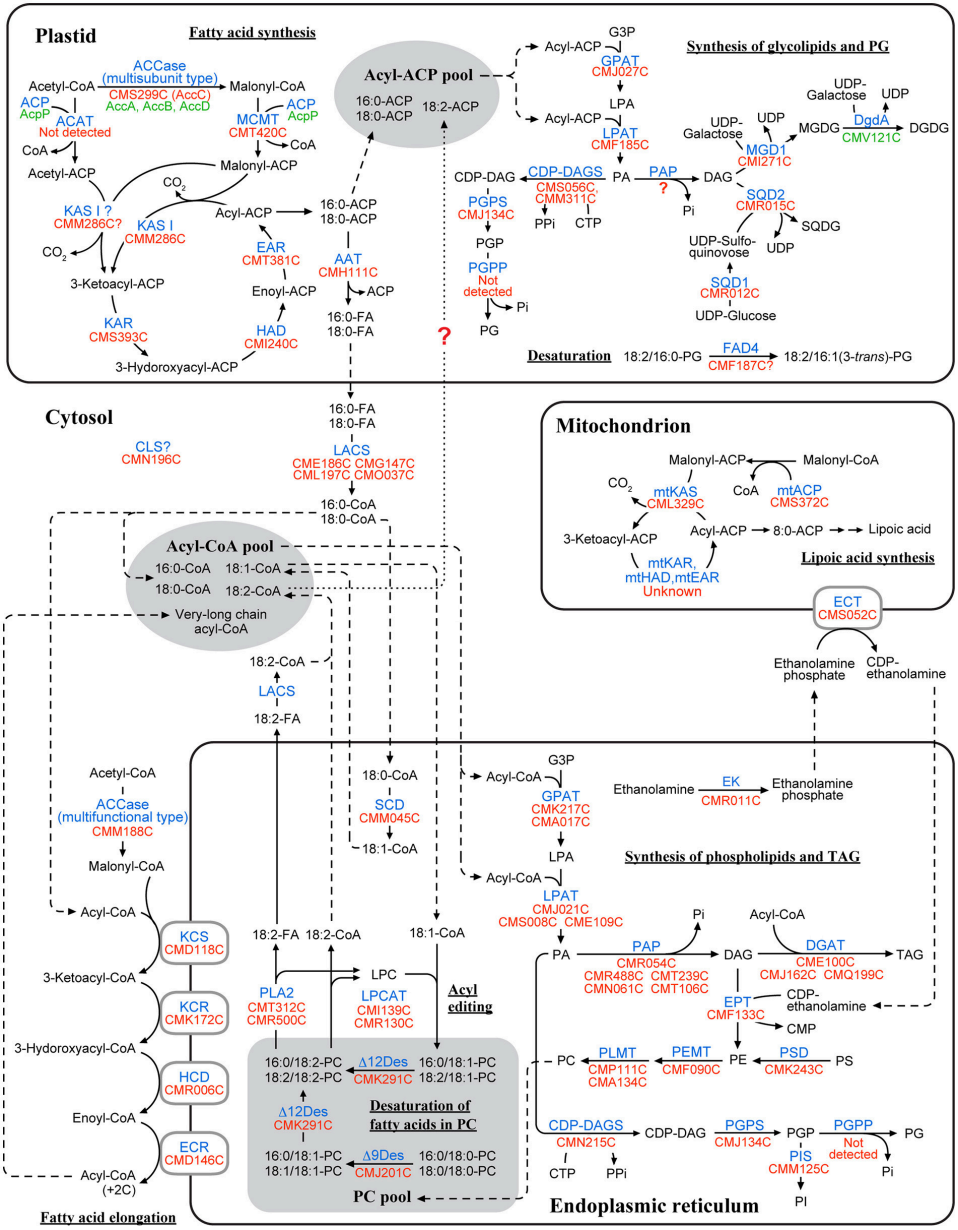
**A map of lipid metabolic pathways in *C. merolae* based on the results of subcellular localization analysis**. This pathway map was created based on the results of subcellular localization analysis (Figure 1D, Supplementary Figures 2–6). Enzyme name is indicated in blue. Locus tag of the genes encoded in the nuclear and the plastid genomes are shown in red and green, respectively. Fatty acids are represented by a combination of the number of carbon atom [X] and the number of double bonds [Y], such as X:Y. The number in the parenthesis indicates position of double bonds. Dashed line indicates flow of transport of substrates. In the plastidic fatty acid synthesis, it is possible that acetyl-CoA:ACP acetyltransferase (ACAT) converts acetyl-CoA to acetyl-ACP in the initial condensation reaction (see Discussion), although putative ACAT was not detected from the genomic data in *C. merolae*. Some enzymes related to mitochondrial fatty acid synthesis, mitochondrial enoyl-CoA reductase (mtEAR), mitochondrial 3-hydroxyacyl-ACP dehydratase (mtHAD), and mitochondrial 3-ketoacyl-ACP reductase (mtKAR), have not been identified either in plants or in *C. merolae*. In the synthesis of glycolipids and PG, phosphatidylglycerolphosphate phosphatase was not detected in the genomic data of *C. merolae*. No PAP candidates were localized in the plastid in *C. merolae*. Putative FAD4 enzyme is included in this figure, although its localization could not be experimentally determined. In *C. merolae*, linoleoyl-CoA is synthesized in the ER, and then is transferred to the plastid, but this transport system is largely unknown (dotted lines). Abbreviations: acyl-ACP, acyl-acyl carrier protein; acyl-CoA, acyl-coenzyme A; CDP-DAG, CDP-diacylglycerol; DAG, diacylglycerol; DGDG, digalactosyldiacylglycerol; FA, fatty acid; G3P, glycerol-3-phosphate; LPA, lysophosphatidic acid; LPL, lysophospholipid; MGDG, monogalactosyldiacylglycerol; PA, phosphatidic acid; PC, phosphatidylcholine; PE, phosphatidylethanolamine; PG, phosphatidylglycerol; PGP, phosphatidylglycerolphosphate; PI, phosphatidylinositol; PL, phospholipid; PS, phosphatidylserine; SQDG, sulfoquinovosyldiacylglycerol; TAG, triacylglycerol. Abbreviated names of enzyme and genes are shown according to Table 1, Supplementary Table 3.

In *C. merolae*, 14 proteins related to fatty acid synthesis were detected by the Gclust analysis (Table 1). Most of these enzymes, namely MCMT (CMT420C), 3-ketoacyl-ACP reductase (KAR; CMS393C), 3-hydroxyacyl-ACP dehydratase (HAD; CMI240C), enoyl-ACP reductase (EAR; CMT381C) and acyl-ACP thioesterase (AAT; CMH111C), were localized in the plastid (Figure 1D, Supplementary Figure 2A). Plastidic ACP is encoded in the plastid genome, and was not analyzed. In addition, *C. merolae* has two types of acetyl-CoA carboxylases (ACCases), namely multifunctional type and multisubunit type, as in plants. Multifunctional type ACCase (ACC1; CMM188C) was localized in the cytosol (Figure 1D). A product of nuclear *AccC* gene (CMS299C) encoding the subunit C of multisubunit type ACCase was targeted to the plastid (Supplementary Figure 2A). Other subunits of multisubunit type ACCase (subunits A, B, and D) are encoded in the plastid genome, so the multisubunit type ACCase is identified as a plastid enzyme. CMM286C and CML329C were identified as KAS, condensing enzymes in the fatty acid synthesis. Analysis of subcellular localization indicated that CMM286C (KAS I) was targeted to the plastid, whereas CML329C (mtKAS) was localized in the mitochondrion (Supplementary Figure 2A). Additionally, *C. merolae* has a mitochondrial ACP (mtACP; CMS372C), which was targeted to the mitochondrion (Supplementary Figure 2A). These results show that *C. merolae* has fatty acid synthesis pathway in both the plastid and the mitochondrion (Figure 2), as in plants.

Because subcellular localization of stearoyl-CoA desaturase (SCD; CMM045C) was determined by GFP-fusion techniques with onion epidermal cells in a previous study (Sato and Moriyama, 2007), we confirmed it by analysis in *C. merolae* cells. There are two codons as candidates of initiator methionine in SCD. A polypeptide starting from the first methionine was localized in the cytosol, whereas a polypeptide starting from the second methionine was targeted to the ER (Supplementary Figure 2B), confirming that SCD is an ER-localized enzyme.

*C. merolae* has seven enzymes involved in fatty acid elongation (Table 1). KCS (CMD118C), 3-ketoacyl-CoA reductase (KCR; CMK172C), 3-hydroxyacyl-CoA dehydratase (HCD; CMR006C), and enoyl-CoA reductase (ECR; CMD146C) were localized in the ER (Supplementary Figure 2C). These results show that fatty acid elongation in *C. merolae* probably takes place in the ER (Figure 2), as in plants. *A. thaliana* has homologs of yeast *ELO* gene encoding KCS enzymes, but the function of most of them remains unclear (Quist et al., 2009; Haslam and Kunst, 2013). Three *ELO-like* homologs (CMT175C, CMM126C, and CML178C) were detected in the genomic data of *C. merolae*. Both CMT175C and CMM126C were targeted to the ER (Supplementary Figure 2C). We were not able to determine the subcellular localization of CML178C, because repeated attempts to detect fluorescence signals were unsuccessful. The *N*-terminus of CML178C was longer than that of the other two homologs, and contained hydrophilic residues at its extremity. The failure of detection of GFP expression for the construct CML178C could be due to inefficient translation or accelerated degradation of the protein product.

### Synthesis of glycolipids and PG

In *C. merolae*, 10 enzymes involved in the synthesis of glycolipids and PG were identified by the Gclust analysis (Table 1). Most of these enzymes, namely lysophosphatidic acid acyltransferase (LPAT; CMF185C), UDP-sulfoquinovose synthase (SQD1; CMR012C), sulfoquinovosyldiacylglycerol synthase (SQD2; CMR015C) and CDP-diacylglycerol synthase (CDP-DAGS; CMS056C and CMM311C), were localized in the plastid (Supplementary Figure 2D). In plastidic CDP-DAGSs, CMM311C was not analyzed, because the amino acid sequence of this protein is identical to that of CMS056C. Phosphatidylglycerolphosphate synthase (PGPS; CMJ134C) was dually localized in the plastid and cytosol (Supplementary Figure 2D). No PAP candidates were targeted to the plastid. DGDG synthase is encoded by a homolog of cyanobacterial *dgdA* gene in the plastid genome (Sakurai et al., 2007), and was not analyzed. Glycerol-3-phosphate acyltransferase (GPAT; CMJ027C) was targeted to the plastid, as evidenced by the HA tag-fusion techniques (Supplementary Figure 2D). *C. merolae* has both enzymes of a plant type monogalactosyldiacylglycerol synthase (MGD1; CMI271C) and an MgdA homolog (CMT267C) that is one of the enzymes involved in MGDG synthesis in cyanobacteria. MGD1 was localized in the plastid, but the MgdA homolog was targeted to the ER (Supplementary Figure 2D). As the epimerase gene *MgdE* was not detected in *C. merolae*, the function of MgdA, if any, might not be related to the synthesis of MGDG (Sato and Awai, 2016). It is likely that MGDG synthesis is performed by MGD1 in *C. merolae*.

These results suggest that the plastid is the site of synthesis of glycolipids and PG in *C. merolae* (Figure 2), as in plants. No homolog of *SFR2* gene encoding galactolipid:galactolipid galactosyltransfrase (Moellering et al., 2010) was detected in the genomic data of *C. merolae*. Recently, phosphatidylglycerolphosphate phosphatase has been identified in plants and green algae (Hung et al., 2015), but no homolog of this enzyme was detected in *C. merolae*. CMF187C was detected as a putative FAD4 protein catalyzing the synthesis of 16:1 (3-*trans*) in plastid PG (Gao et al., 2009). We detected this unsaturated fatty acid (Sato and Moriyama, 2007; Toyoshima et al., 2016), but, unfortunately, we were not able to detect the signal of CMF187C within the cell by repeated attempts.

### Synthesis of phospholipids and TAG

In *C. merolae*, 27 enzymes involved in the synthesis of phospholipids and TAG were detected by the Gclust analysis (Table 1). Most of these enzymes, for example GPAT (CMK217C and CMA017C), phosphatidylinositol synthase (PIS; CMM125C) and DGAT (CMQ199C, CME100C and CMJ162C), were targeted to the cytosol or ER (Figure 1D, Supplementary Figure 3). ECT was localized in the mitochondrion, as in plants (Mizoi et al., 2006; Figure 1D). No gene encoding phosphatidylserine synthase was detected in the genomic data of *C. merolae*, a fact which is consistent with the inability of detection of PS in *C. merolae* cells (Sato and Moriyama, 2007). In the same study, subcellular localizations of acyl-lipid Δ9 desaturase (Δ9Des; CMJ201C) and acyl-lipid Δ12 desaturase (Δ12Des; CMK291C) were analyzed by GFP-fusion techniques with onion epidermal cells (Sato and Moriyama, 2007), resulting in inconsistent localization depending on the selection of initiation codon. We now analyzed the subcellular localization of these desaturases in *C. merolae* cells. In Δ9Des, both polypeptides starting from the first and the second methionines were localized in the ER (Supplementary Figure 2B). In Δ12Des, both polypeptides starting from the first and the third methionines were localized in the ER, while a polypeptide starting from the second methionine was targeted to the cytosol (Supplementary Figure 2B). Based on these results, we consider that Δ9Des and Δ12Des are ER-localized enzymes. All these results suggest that the major site of synthesis of phospholipids and TAG must reside in the ER in *C. merolae* (Figure 2). This is a situation quite similar to that in plants, in contrast with mis-tuned targeting predictions (Table 1) and the results in heterologous expression systems.

In plants, PC is synthesized by both CDP-choline pathway and PE methylation pathway. However, *C. merolae* has no enzymes involved in the CDP-choline pathway, such as choline kinase (CK), phosphocholine cytidylyltransferase (CCT) and phosphoethanolamine methyltransferase (PEAMT). This suggests that PE methylation is the only identifiable pathway of PC synthesis in *C. merolae* (Figure 2), which was confirmed by labeling experiments using [^32^P] phosphate (Sato et al., 2016b).

### **β**-oxidation

Nine enzymes related to β-oxidation of fatty acids were found in *C. merolae* by the Gclust analysis (Table 1). Four enzymes were localized in the mitochondrion, and CMC137C encoding 3-hydroxyacyl-CoA dehydrogenase (HACDH) was dually localized to the cytosol and mitochondrion (Supplementary Figure 4A). Other β-oxidation enzymes were targeted to the plastid or cytosol (Supplementary Figure 4A). Because it is possible that non-mitochondrial or non-plastid enzymes, namely acyl-CoA oxidase (ACX; CMK115C), multifunctional protein (MFP; CMR380C) and 3-ketoacyl-CoA thiolase (KAT; CMA042C), might be localized in the peroxisome, subcellular localization of these enzymes were analyzed with constructs of GFP-fused *C*-terminal peptide (Supplementary Figure 4B). These enzymes, however, were not localized in the peroxisome in any constructs.

**Figure 3:**
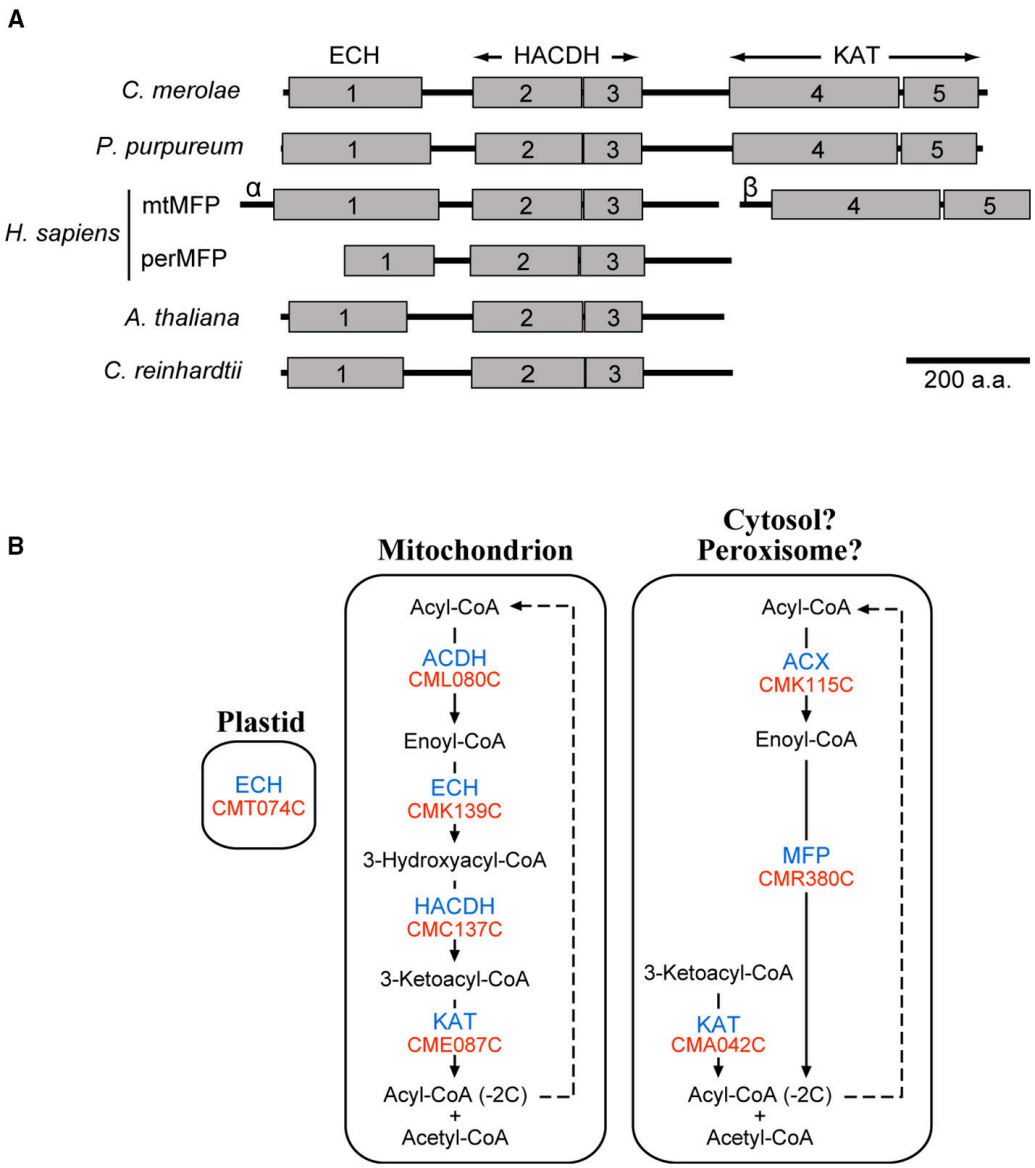
**β-Oxidation of fatty acids in *C. merolae***. **(A)** Schematic domain structure of representative MFPs. We searched for functional domains of these MFPs using the Pfam database (Finn et al., 2014). Gray boxes indicate individual functional domains, namely enoyl-CoA hydratase/isomerase family domain (1), 3-hydroxyacyl-CoA dehydrogenase NAD binding domain (2), 3-hydroxyacyl-CoA dehydrogenase *C*-terminal domain (3), thiolase *N*-terminal domain (4), and thiolase *C*-terminal domain (5). *Homo sapience* has both peroxisomal MFP (perMFP) and mitochondrial MFP (mtMFP). mtMFP is a complex consisting of α and β subunits (α or β). **(B)** A pathway map of β-oxidation in *C. merolae*. This pathway map was created based on the results of subcellular localization analysis (Supplementary Figures 4A,B). For abbreviated names of enzymes and genes, see Table 1.

Figure 3A indicates schematic domain structure of MFPs in various organisms. Peroxisomal MFP in plants and animals has both enoyl-CoA hydratase (ECH) and HACDH domains. In animals, mitochondrial MFP forms a heterooctamer complex consisting of α and β subunits, of which the former subunit has ECH and HACDH domains, whereas a KAT domain exists in the latter subunit. By using the Pfam database (Finn et al., 2014), we found that *C. merolae* MFP has ECH, HACDH, and KAT domains within a single polypeptide chain. This suggests that *C. merolae* MFP might catalyze the three successive reactions of β-oxidation against saturated acyl-CoA as a substrate. The domain structure of *C. reinhardtii* MFP was similar to that of plant homologs. *Porphyridium purpureum*, a red alga belonging to a different class from *C. merolae* contains an MFP, whose domain structure was similar to that in *C. merolae* MFP. It is likely that MFP having three domains in a single polypeptide chain is one of the features in red algae. Therefore, these results showed that *C. merolae* might have two β-oxidation pathways consisting of mitochondrial- and extra-mitochondrial-enzymes (Figure 3B).

### Other enzymes related to acyl lipid metabolism

Results of subcellular localization analysis of enzymes related to lipid degradation, lipid trafficking, fatty acid activation, PI signaling, and biotin-dependent carboxylation are reported in Supplementary Data 1, and summarized in Supplementary Table 3.

## Discussion

In the present study, we attempted to identify all the enzymes involved in acyl lipid metabolism in *C. merolae* and to analyze their subcellular localization as an initial basis for the future biochemical and genetic studies. We detected 121 enzymes involved in the metabolism of acyl lipids, and identified the subcellular localization of 110 of the enzymes by experimental analysis. These results suggest that the roles of different compartments in the synthesis of fatty acids and lipids are largely similar in *C. merolae* to those in plants, in spite of the strange predictions by various software, showing diversified localization. Table 2 indicates a comparison of the predictions of these computer programs with the results of subcellular localization analysis using the GFP- or HA tag-fusion techniques. Most of performance scores were less than 0.6. It seems likely that prediction of proteins localized in the plastid by TargetP was correct at a high rate (performance score; 0.72), but approximately half of the plastidic enzymes confirmed by experimental analysis were predicted as mitochondrial proteins by this program. The same is true of proteins in the secretory pathway. Although, they had a high performance score for secretory pathway proteins, TargetP and PredAlgo mispredicted proteins localized in the ER and/or the cytoplasmic membrane as target to mitochondrion and cytosol. These results suggest that these prediction tools for plants and green algae were not suitable for proteins in *C. merolae*, because they are not tuned or trained for the red alga. We consider that this is due to the fact that the targeting signals in this alga could be significantly different from those in both plants and green algae. In this respect, the results of the present study, as well as the results of a previous study (Moriyama et al., 2014a), will be useful as a training set for the neural network used in the prediction tools.

**Table 2:** **Poor performance of various prediction tools with respect to the localization of *C. merolae* proteins**.

**Computer program**	**Prediction**	**Confirmed subcellular localization**				**Performance score**
		**Pt**	**Mt**	**ER and/or CM**	**Cyt**	
TargetP	Pt (18)	13	1	3	1	0.72
	Mt (38)	10	11	15	2	0.29
	SP (7)	0	0	5	2	0.71
	Other (43)	4	2	22	15	0.35
WoLF PSORT	Pt (44)	16	8	14	6	0.36
	Mt (7)	2	2	3	0	0.29
	ER, PM (27)	4	0	16	7	0.59
	Cyt (12)	3	1	3	5	0.42
PredAlgo	Pt (27)	14	3	8	2	0.52
	Mt (17)	4	4	6	3	0.24
	SP (16)	1	2	13	0	0.81
	Other (46)	8	5	18	15	0.33

In the results of prediction using TargetP and PredAlgo, we considered that “SP” (secretory pathway) means targeting to the ER and/or the cytoplasmic membrane, and “Other” means targeting to the cytosol. In the results of WoLF PSORT, some enzymes predicted to localize in more than two compartments were not included in this rating. Abbreviations are listed in the legend for Table 1. Performance score is the proportion of correct localization.

In this study, we constructed the acyl lipid metabolic map in *C. merolae* (Figure 2) based on comparative genomics and subcellular localization analysis, and found notable characteristics of lipid metabolism in this alga. However, homology-based identification has limitation in that they cannot theoretically predict detailed characteristics of the enzymes. Additional biochemical or genetic analyses will be required for elaborating this map. We discuss on the following topics: First, only a single enzyme KAS I being involved in the condensation reaction of fatty acid synthesis in the plastid. Second, pathways of TAG synthesis in *C. merolae*. Third, the sites of β-oxidation pathways in *C. merolae*. All these points will be important research projects for future biochemical and genetic studies.

### A single KAS enzyme catalyzes condensation reactions in plastidic fatty acid synthesis

We found KAS I and mtKAS, but not KAS II or KAS III, in *C. merolae* (Figure 2). Since condensation reactions of mitochondrial fatty acid synthesis are catalyzed solely by mtKAS in plants (Yasuno et al., 2004), it is expected that mitochondrial fatty acid synthesis in *C. merolae* might be catalyzed by a similar system. Plastidic fatty acid synthesis in plants involves three isoforms of KAS having different substrate specificities. Initial condensation reaction is catalyzed by KAS III, which catalyzes the formation of acetoacetyl-ACP from acetyl-CoA and malonyl-ACP (Clough et al., 1992). Subsequent condensations up to palmitoyl-ACP are catalyzed by KAS I, whereas the final elongation of palmitoyl-ACP to stearoyl-ACP requires KAS II (Shimakata and Stumpf, 1982; Pidkowich et al., 2007). Likewise, three isoforms of KAS participate in the fatty acid synthesis in *Escherichia coli* and cyanobacteria (Lem and Stumpf, 1984; Parsons and Rock, 2013). Because *C. merolae* lacks KAS II and KAS III, KAS I alone is supposed to catalyze all condensation reactions of fatty acid synthesis in the plastid. We tried to look for an alternative enzyme, acetyl-CoA:ACP acetyltransferase (ACAT), which could convert acetyl-CoA to acetyl-ACP in the initial condensation reaction, normally as part of the type I fatty acid synthase in animals and yeasts (Smith et al., 2003; Tehlivets et al., 2007), but we were not able to detect a homolog. As KAS I/II and KAS III are distinguished by functional domains in plants and bacteria, KAS III cannot be replaced by KAS I there. In Gram-negative pathogen *Pseudomonas aeruginosa*, FabY plays as an initial condensing enzyme in fatty acid synthesis, though it has a KAS I/II domain (Yuan et al., 2012). It is quite possible that *C. merolae* KAS I is able to catalyze the initial condensation reaction like FabY. Another question is related to the end product of fatty acid synthesis in *C. merolae*, if the final elongation from C16 to C18 is not catalyzed by KAS II. These points should be clarified by biochemical analysis using the plastids or isolated KAS I protein.

### Pathway of TAG synthesis in *C. merolae*

The simplest pathway of *de novo* TAG synthesis consists of four steps, the two acylations of glycerol-3-phosphate (G3P) by GPAT and LPAT, the dephosphorylation of phosphatidic acid by PAP, and the third acylation of DAG by DGAT or phospholipid:diacylglycerol acyltransferase (PDAT). PC is a key intermediate in the synthesis of TAG having polyunsaturated fatty acids, because PC is a major substrate of acyl lipid desaturase in ER (Bates et al., 2013). Transferring a polyunsaturated fatty acid from PC to DAG by PDAT is an important pathway of TAG synthesis via PC in plants. Phosphatidylcholine:diacylglycerol cholinephosphotransferase (PDCT) catalyzes transfer of a phosphocholine head group from PC to DAG, which is another important enzyme related to TAG synthesis via PC (Lu et al., 2009). This enzyme is able to produce DAG from unsaturated PC. PC-derived DAG are also produced by the degradation by phospholipase C (PLC) or phospholipase D (PLD) plus PAP and the reverse reaction of CPT. Genes encoding PDAT, PDCT, PLC, and PLD were not detected in *C. merolae* (Table 1, Supplementary Table 3). These results suggest that TAG is synthesized without the production of PC-derived DAG in *C. merolae*, although a large amount of TAG containing linoleic acid was detected in *C. merolae* by lipid analysis (Toyoshima et al., 2016). TAG could be produced by acylation of the DAG, which is formed from acyl CoA pool in equilibrium with the acyl groups in PC. This point will be another future project involving the metabolic flow of fatty acids in TAG synthesis in *C. merolae*.

### The sites of **β**-oxidation of fatty acids in *C. merolae*

The results showed that *C. merolae*, unlike plants, might have two β-oxidation pathways consisting of mitochondrial- and extra-mitochondrial-enzymes (Figure 3B). The subcellular localization of the extra-mitochondrial β-oxidation enzymes, namely ACX, MFP, and KAT (CMA042C), were examined using the constructs of EGFP-fused *C*-terminal peptides of these enzymes, which were not localized in the peroxisome (Supplementary Figure 4B). No peroxisomal targeting signals (Lingner et al., 2011) were detected from β-oxidation enzymes except for ACX in *C. merolae*. Additionally, these enzymes were not detected by proteome analysis of isolated *C. merolae* peroxisomes (Imoto et al., 2013). These might suggest that the sites of extra-mitochondrial β-oxidation pathways might be in the cytosol in *C. merolae*, as in bacteria. This should be investigated experimentally, because this is an unprecedented situation.

## Author contributions

All authors listed, have made substantial, direct and intellectual contribution to the work, and approved it for publication.

### Conflict of interest statement

The authors declare that the research was conducted in the absence of any commercial or financial relationships that could be construed as a potential conflict of interest.
