# Supplementary material for: Construction of Global Acyl Lipid Metabolic Map by Comparative Genomics and Subcellular Localization Analysis in the Red Alga Cyanidioschyzon merolae
Source: Front Plant Sci. 2016 Jun 30;7:958. doi: 10.3389/fpls.2016.00958 (PMC4928187; doi:10.3389/fpls.2016.00958)
Supplement: Supplementary file 1 [file DataSheet1.pdf]

frontiers

Supplementary Material

Construction of Global Acyl Lipid Metabolic Map by Comparative Genomics and Subcellular Localization Analysis in the Red Alga *Cyanidioschyzon merolae*

Natsumi Mori, Takashi Moriyama, Masakazu Toyoshima, Naoki Sato*

*** Correspondence:** Naoki Sato: naokisat@bio.c.u-tokyo.ac.jp

1 Supplementary Data

1.1 Supplementary Data 1. Results of Subcellular Localization Analysis of Other Enzymes Related to Acyl Lipid Metabolism in *C. merolae*

1.1.1 Lipase

By comparative genomic analysis, 20 enzymes involved in lipid degradation were detected in the genomic data of *C. merolae* (Supplementary Table 3). Eight enzymes were putative triacylglycerol lipases (TAGL). Six enzymes were also found as hydrolases that might act as lipases. Most of putative TAGLs and hydrolases were targeted to the cytosol or ER, but two TAGLs (CMP157C and CMR088C) and a hydrolase (CMT308C) were localized in the plastid (Supplementary Figure 5A), suggesting that these enzymes might be involved in the degradation of plastid membrane lipids. Additionally, *C. merolae* has three phospholipases A1 (PLA1; CMP267C, CMH204C and CMQ413C) and two phospholipases A2 (PLA2; CMR500C and CMT312C). In PLA1, CMP267C and CMH204C were targeted to the cytosol, whereas the fluorescence of GFP-fused CMQ413C was observed in both the cytoplasmic membrane and vesicle (Supplementary Figure 5A). *C. merolae* PLA1s are similar to the putative PA-preferring PLA1 in *A. thaliana* (Kato et al., 2002). In PLA2, CMR500C showed dual localization to the plastid and the nucleus, whereas CMT312C was localized in both the plastid and the ER (Supplementary Figure 5A). It seems that these enzymes are components of the acyl editing cycle (Lager et al., 2013; Pan et al., 2015) with lysophospholipid acyltransferase (LPLAT) encoded by CMI139C and CMR130C (Supplementary Figure 3). The existence of acyl editing cycle in *C. merolae* is supported by the finding that radioactive carbons were quickly incorporated into PC (Sato and Moriyama, 2007).

1.1.2 Lipid Trafficking

It is thought that TGD1-3 complex and TGD4 function as lipid transporters in the plastid envelopes (Hurlock et al., 2014). *C. merolae* has putative *TGD1* and *TGD2* homologs encoded in the plastid genome, and four putative *TGD3* homologs (CMR180C, CMH235C, CMJ039C, and CMR388C), whereas no *TGD4* homologs were detected (Supplementary Table 3). By subcellular localization analysis, CMR180C, CMJ039C and CMH235C were targeted to the ER, but CMR388C was

localized in the plastid (Supplementary Figure 5B). It is likely that CMR388C is the *TGD3* ortholog in *C. merolae*.

Acyl-CoA binding proteins (ACBP) are thought to transfer fatty acids from the plastid to the ER in plants (Xiao and Chye, 2011). In *A. thaliana*, six isoforms, ACBP1-6, have been identified. *C. merolae* has a gene encoding *ACBP6* ortholog (CMP278C, Supplementary Table 3). *A. thaliana* ACBP6 is a cytosolic, smallest ACBP (Xiao and Chye, 2011). *C. merolae* ACBP was also localized in the cytosol (Supplementary Figure 5B).

Flippase catalyzes the movement of polar lipids between the two membrane leaflets that does not happen spontaneously, which finally results in asymmetric distribution of lipids between the two leaflets of a membrane. In *A. thaliana*, the P4 subfamily of ATPases, ALA1-12, are believed to act as flippases (Gomès et al., 2000). Among them, ALA1-3 already have been characterized (López-Marqués et al., 2010, 2012; Poulsen et al., 2008). Additionally, ALA-interacting subunit (ALIS) proteins are involved in the determination of subcellular localization of ALA enzymes (López-Marqués et al., 2010, 2012; Poulsen et al., 2008). In *C. merolae*, two flippases (ALA1; CMR306C and ALA2; CMS375C) and an ALIS protein (CMT246C) were detected by the Gclust analysis (Supplementary Table 3). Both ALA1 and ALIS proteins were dually localized to the ER and cytoplasmic membrane (Figure 1D and Supplementary Figure 5B). ALA2 was targeted to the nucleus and cytosol (Supplementary Figure 5B).

1.1.3 Fatty Acid Activation, PI Signaling and Biotin-Dependent Carboxylation

Two genes encoding acyl-CoA thioesterases (ACT; CMJ263C and CMR113C) and five genes encoding long-chain acyl-CoA synthetases (LACS; CME186C, CMG147C, CMO037C, CML197C and CMT459C) were found in the genomic data of *C. merolae* (Supplementary Table 3). Both ACT enzymes were targeted to the mitochondrion (Supplementary Figure 6A). In LACSs, CME186C, CMG147C, CMO037C were localized in the cytosol, but CML197C showed dual localization to the cytosol and plastid (Supplementary Figure 6A). GFP-fused CMT459C was localized in the plastid (Supplementary Figure 6A).

C. merolae has two each genes encoding phosphatidylinositol-4-kinases (PI4K; CMI125C and CMS267C) and phosphatidylinositol-4-phosphate 5-kinases (PIP5K; CMN333C and CME153C), which are involved in the PI signaling (Supplementary Table 3). CMI125C and CMN333C were localized in the ER, whereas CMS267C and CME157C were targeted to both cytosol and cytoplasmic membrane (Supplementary Figure 6B).

Biotin is attached to biotin-dependent enzymes, such as carboxylases or decarboxylases, by posttranslational modification catalyzed by holocarboxylase synthetase (HCS). In *C. merolae,* HCS (CMC080C) was dually localized in the plastid and cytosol, as in plants (Supplementary Figure 6C). Additionally, we analyzed subcellular localization of typical biotin-dependent enzymes, namely methylcrotonyl-CoA carboxylase (MCC), propionyl-CoA carboxylase (PCC) and carbamoylphosphate synthase (CAR). Individual subunits of MCC (MCCA; CMT073C and MCCB; CMT071C) and PCC (PCCA; CMN243C and PCCB; CMM132C) were localized in the mitochondrion (Supplementary Figure 6C). *C. merolae* has two types of CARs involved in pyrimidine synthesis and arginine synthesis. They are multifunctional type and multisubunit type, respectively. Subcellular localization of a multifunctional type CAR (CAR1; CMQ255C) was not examined yet. In multisubunit type CAR, the small subunit (CarA) is encoded in the plastid genome,

and was not analyzed. A product of nuclear *CarB* gene (CML055C) encoding the large subunit of CAR was targeted in the plastid as expected (Supplementary Figure 6C).

1.1.4 References

Gomès, E., Jakobsen, M. K., Axelsen, K. B., Geisler, M., and Palmgren, M. G. (2000). Chilling tolerance in Arabidopsis involves ALA1, a member of a new family of putative aminophospholipid translocases. *Plant Cell* 12, 2441–2454. doi:10.1105/tpc.12.12.2441.

Hurlock, A. K., Roston, R. L., Wang, K., and Benning, C. (2014). Lipid trafficking in plant cells. *Traffic* 15, 915–932. doi:10.1111/tra.12187.

Kato, T., Morita, M. T., Fukaki, H., Yamauchi, Y., Uehara, M., Niihama, M., et al. (2002). SGR2, a phospholipase-like protein, and ZIG/SGR4, a SNARE, are involved in the shoot gravitropism of Arabidopsis. *Plant Cell* 14, 33–46. doi:10.1105/tpc.010215.

Lager, I., Yilmaz, J. L., Zhou, X. R., Jasieniecka, K., Kazachkov, M., Wang, P., et al. (2013). Plant acyl-CoA:lysophosphatidylcholine acyltransferases (LPCATs) have different specificities in their forward and reverse reactions. *J. Biol. Chem.* 288, 36902–36914. doi:10.1074/jbc.M113.521815.

López-Marqués, R. L., Poulsen, L. R., Hanisch, S., Meffert, K., Buch-Pedersen, M. J., Jakobsen, M. K., et al. (2010). Intracellular targeting signals and lipid specificity determinants of the ALA/ALIS P_4-ATPase complex reside in the catalytic ALA α-subunit. *Mol. Biol. Cell* 21, 791–801. doi:10.1091/mbc.E09-08-0656.

López-Marqués, R. L., Poulsen, L. R., and Palmgren, M. G. (2012). A putative plant aminophospholipid flippase, the arabidopsis P4 ATPase ALA1, localizes to the plasma membrane following association with a β-subunit. *PLoS One* 7, e33042. doi:10.1371/journal.pone.0033042.

Pan, X., Chen, G., Kazachkov, M., Greer, M. S., Caldo, K. M. P., Zou, J., et al. (2015). *In vivo* and *In vitro* Evidence for biochemical coupling of reactions catalyzed by lysophosphatidylcholine acyltransferase and diacylglycerol acyltransferase. *J. Biol. Chem.* 290, jbc.M115.654798. doi:10.1074/jbc.M115.654798.

Poulsen, L. R., López-Marqués, R. L., McDowell, S. C., Okkeri, J., Licht, D., Schulz, A., et al. (2008). The *Arabidopsis* P_4-ATPase ALA3 localizes to the golgi and requires a β-subunit to function in lipid translocation and secretory vesicle formation. *Plant Cell* 20, 658–676. doi:10.1105/tpc.107.054767.

Sato, N., and Moriyama, T. (2007). Genomic and biochemical analysis of lipid biosynthesis in the unicellular rhodophyte *Cyanidioschyzon merolae*: Lack of a plastidic desaturation pathway results in the coupled pathway of galactolipid synthesis. *Eukaryot. Cell* 6, 1006–1017. doi:10.1128/EC.00393-06.

Xiao, S., and Chye, M. L. (2011). New roles for acyl-CoA-binding proteins (ACBPs) in plant development, stress responses and lipid metabolism. *Prog. Lipid Res.* 50, 141–151. doi:10.1016/j.plipres.2010.11.002.

1.2 Supplementary Data 2. List of References Cited in Supplementary Table 2.

Arent S, Christensen CE, Pye VE, Nørgaard A, Henriksen A (2010) The multifunctional protein in peroxisomal β-oxidation: Structure and substrate specificity of the *Arabidopsis thaliana* protein MFP2. J Biol Chem 285: 24066–24077

Awai K, Kakimoto T, Awai C (2006) Comparative genomic analysis revealed a gene for monoglucosyldiacylglycerol synthase, an enzyme for photosynthetic membrane lipid synthesis in cyanobacteria. Plant Physiol 141: 1120–1127

Awai K, Maréchal E, Block MA, Brun D, Masuda T, Shimada H, Takamiya K, Ohta H, Joyard J (2001) Two types of MGDG synthase genes, found widely in both 16:3 and 18:3 plants, differentially mediate galactolipid syntheses in photosynthetic and nonphotosynthetic tissues in *Arabidopsis thaliana*. Proc Natl Acad Sci U S A 98: 10960–10965

Awai K, Ohta H, Sato N (2014) Oxygenic photosynthesis without galactolipids. Proc Natl Acad Sci U S A 111: 13571–13575

Awai K, Xu C (2006) A phosphatidic acid-binding protein of the chloroplast inner envelope membrane involved in lipid trafficking. Proc Natl Acad Sci U S A 2: 1–6

Aymé L, Baud S, Dubreucq B, Joffre F, Chardot T (2014) Function and localization of the *Arabidopsis thaliana* diacylglycerol acyltransferase DGAT2 expressed in yeast. PLoS One 9: e92237

Babiychuk E, Müller F, Eubel H, Braun H-P, Frentzen M, Kushnir S (2003) *Arabidopsis* phosphatidylglycerophosphate synthase 1 is essential for chloroplast differentiation, but is dispensable for mitochondrial function. Plant J 33: 899–909

Bach L, Michaelson L (2008) The very-long-chain hydroxy fatty acyl-CoA dehydratase PASTICCINO2 is essential and limiting for plant development. Proc Natl Acad Sci U S A 105: 14727-14731

Beisson F, Li Y, Bonaventure G, Pollard M, Ohlrogge JB (2007) The Acyltransferase GPAT5 Is Required for the Synthesis of Suberin in Seed Coat and Root of *Arabidopsis*. Plant Cell 19: 351–368

Bolognese C, McGraw P (2000) The Isolation and Characterization in Yeast of a Gene for Arabidopsis *S*-Adenosylmethionine: Phospho-Ethanolamine *N*-Methyltransferase. Plant Physiol 124: 1800–1813

Bouvier-Navé P, Benveniste P, Oelkers P, Sturley SL, Schaller H (2000) Expression in yeast and tobacco of plant cDNAs encoding acyl CoA:diacylglycerol acyltransferase. Eur J Biochem 267: 85–96

Camacho L, Smertenko AP, Perez-Gomez J, Hussey PJ, Moore I (2009) *Arabidopsis* Rab-E GTPases exhibit a novel interaction with a plasma-membrane phosphatidylinositol-4-phosphate 5-kinase. J Cell Sci 122: 4383–4392

La Camera S, Geoffroy P, Samaha H, Ndiaye A, Rahim G, Legrand M, Heitz T (2005) A pathogen-inducible patatin-like lipid acyl hydrolase facilitates fungal and bacterial host colonization in *Arabidopsis*. Plant J 44: 810–825

Cao Z, Zhang J, Li Y, Xu X, Liu G, Bhattacharrya MK, Yang H, Ren D (2007) Preparation of polyclonal antibody specific for AtPLC4, an *Arabidopsis* phosphatidylinositol-specific phospholipase C in rabbits. Protein Expr Purif 52: 306–312

Carrie C, Murcha MW, Millar a H, Smith SM, Whelan J (2007) Nine 3-ketoacyl-CoA thiolases (KATs) and acetoacetyl-CoA thiolases (ACATs) encoded by five genes in *Arabidopsis thaliana* are targeted either to peroxisomes or cytosol but not to mitochondria. Plant Mol Biol 63: 97–108

Cases S, Smith SJ, Zheng YW, Myers HM, Lear SR, Sande E, Novak S, Collins C, Welch CB, Lusis AJ, Erickson SK, Farese RV Jr. (1998) Identification of a gene encoding an acyl CoA:diacylglycerol acyltransferase, a key enzyme in triacylglycerol synthesis. Proc Natl Acad Sci U S A 95: 13018–13023

Chen HH, Wickrema A, Jaworski JG (1988) Acyl-acyl-carrier protein: lysomonogalactosyldiacylglycerol acyltransferase from the cyanobacterium *Anabaena variabilis*. Biochim Biophys Acta 963: 493–500

Chen M, Thelen JJ (2013) ACYL-LIPID DESATURASE2 is required for chilling and freezing tolerance in *Arabidopsis*. Plant Cell 25: 1430–1444

Choi YH, Lee JK, Lee CH, Cho SH (2000) cDNA cloning and expression of an aminoalcoholphosphotransferase isoform in Chinese cabbage. Plant Cell Physiol 41: 1080–1084

Chrost B, Kolukisaoglu U, Schulz B, Krupinska K (2007) An alpha-galactosidase with an essential function during leaf development. Planta 225: 311–320

Clough RC, Matthis AL, Barnum SR, Jaworski JG (1992) Purification and characterization of 3-ketoacyl-acyl carrier protein synthase III from spinach. A condensing enzyme utilizing acetyl-coenzyme A to initiate fatty acid synthesis. J Biol Chem 267: 20992–20998

Collin S, Justin AM, Cantrel C, Arondel V, Kader JC (1999) Identification of AtPIS, a phosphatidylinositol synthase from *Arabidopsis*. Eur J Biochem 262: 652–658

Costa M Da, Bach L, Landrieu I (2006) *Arabidopsis* PASTICCINO2 is an antiphosphatase involved in regulation of cyclin-dependent kinase A. Plant Cell 18: 1426–1437

Dahlqvist A, Stahl U, Lenman M, Banas A, Lee M, Sandager L, Ronne H, Stymne S (2000) Phospholipid:diacylglycerol acyltransferase: an enzyme that catalyzes the acyl-CoA-independent formation of triacylglycerol in yeast and plants. Proc Natl Acad Sci U S A 97: 6487–6492

Däschner K, Couée I, Binder S (2001) The Mitochondrial Isovaleryl-Coenzyme A Dehydrogenase of Arabidopsis Oxidizes Intermediates of Leucine and Valine Catabolism. Plant Physiol 126: 601–612

Dewey RE, Wilson RF, Novitzky WP, Goode JH (1994) The *AAPT1* gene of soybean complements a cholinephosphotransferase-deficient mutant of yeast. Plant Cell 6: 1495–1507

Dörmann P, Balbo I, Benning C (1999) *Arabidopsis* galactolipid biosynthesis and lipid trafficking mediated by DGD1. Science 284: 2181–2184

Eastmond PJ (2006) SUGAR-DEPENDENT1 Encodes a Patatin Domain Triacylglycerol Lipase That Initiates Storage Oil Breakdown in Germinating *Arabidopsis* Seeds. Plant Cell 18: 665–675

Eastmond PJ, Hooks MA, Williams D, Lange P, Bechtold N, Sarrobert C, Nussaume L, Graham IA (2000) Promoter trapping of a novel medium-chain acyl-CoA oxidase, which is induced transcriptionally during *Arabidopsis* seed germination. J Biol Chem 275: 34375–34381

Eastmond PJ, Quettier A-L, Kroon JTM, Craddock C, Adams N, Slabas AR (2010) Phosphatidic acid phosphohydrolase 1 and 2 regulate phospholipid synthesis at the endoplasmic reticulum in *Arabidopsis*. Plant Cell 22: 2796–2811

Essigmann B, Güler S, Narang RA, Linke D, Benning C (1998) Phosphate availability affects the thylakoid lipid composition and the expression of *SQD1*, a gene required for sulfolipid biosynthesis in *Arabidopsis thaliana*. Proc Natl Acad Sci U S A 95: 1950–1955

Eubel H, Meyer EH, Taylor NL, Bussell JD, O'Toole N, Heazlewood JL, Castleden I, Small ID, Smith SM, Millar AH (2008) Novel proteins, putative membrane transporters, and an integrated metabolic network are revealed by quantitative proteomic analysis of *Arabidopsis* cell culture peroxisomes. Plant Physiol 148: 1809–1829

Falcone DL, Gibson S, Lemieux B, Somerville C (1994) Identification of a gene that complements an *Arabidopsis* mutant deficient in chloroplast omega 6 desaturase activity. Plant Physiol 106: 1453–1459

Froman BE, Edwards PC, Bursch AG, Dehesh K (2000) ACX3, a novel medium-chain acyl-coenzyme A oxidase from *Arabidopsis*. Plant Physiol 123: 733–742

Fulda M, Shockey J, Werber M (2002) Two long-chain acyl-CoA synthetases from *Arabidopsis thaliana* involved in peroxisomal fatty acid β-oxidation. Plant J 93–103

Gao J, Ajjawi I, Manoli A, Sawin A, Xu C, Froehlich JE, Last RL, Benning C (2009) FATTY ACID DESATURASE4 of *Arabidopsis* encodes a protein distinct from characterized fatty acid desaturases. Plant J 60: 832–839

Germain V, Rylott EL, Larson TR, Sherson SM, Bechtold N, Carde JP, Bryce JH, Graham IA, Smith SM (2001) Requirement for 3-ketoacyl-CoA thiolase-2 in peroxisome development, fatty acid

beta-oxidation and breakdown of triacylglycerol in lipid bodies of *Arabidopsis* seedlings. Plant J 28: 1–12

Ghosh AK, Chauhan N, Rajakumari S, Daum G, Rajasekharan R (2009) At4g24160, a soluble acyl-coenzyme A-dependent lysophosphatidic acid acyltransferase. Plant Physiol 151: 869–881

Gidda SK, Shockey JM, Rothstein SJ, Dyer JM, Mullen RT (2009) *Arabidopsis thaliana* GPAT8 and GPAT9 are localized to the ER and possess distinct ER retrieval signals: functional divergence of the dilysine ER retrieval motif in plant cells. Plant Physiol Biochem 47: 867–879

Goepfert S (2005) Molecular Identification and Characterization of the *Arabidopsis* 3,5, 2,4-Dienoyl-Coenzyme A Isomerase, a Peroxisomal Enzyme Participating in the β-Oxidation Cycle of Unsaturated Fatty Acids. Plant Physiol 138: 1947–1956

Goepfert S, Hiltunen JK, Poirier Y (2006) Identification and Functional Characterization of a Monofunctional Peroxisomal Enoyl-CoA Hydratase 2 That Participates in the Degradation of Even cis-Unsaturated Fatty Acids in *Arabidopsis thaliana*. J Biol Chem 281: 35894–35903

Goepfert S, Vidoudez C, Tellgren-Roth C, Delessert S, Hiltunen JK, Poirier Y (2008) Peroxisomal Delta(3),Delta(2)-enoyl CoA isomerases and evolution of cytosolic paralogues in embryophytes. Plant J 56: 728–742

Gomès E, Jakobsen M (2000) Chilling tolerance in *Arabidopsis* involves ALA1, a member of a new family of putative aminophospholipid translocases. Plant Cell 12: 2441–2453

Hagio M, Gombos Z, Várkonyi Z, Masamoto K, Sato N, Tsuzuki M, Wada H (2000) Direct evidence for requirement of phosphatidylglycerol in photosystem II of photosynthesis. Plant Physiol 124: 795–804

Haselier A, Akbari H, Weth A, Baumgartner W, Frentzen M (2010) Two closely related genes of *Arabidopsis* encode plastidial cytidinediphosphate diacylglycerol synthases essential for photoautotrophic growth. Plant Physiol 153: 1372–1384

Haslam TM, Haslam R, Thoraval D, Pascal S, Delude C, Domergue F, Fernández AM, Beaudoin F, Napier JA, Kunst L, Joubés J (2015) ECERIFERUM2-LIKE Proteins Have Unique Biochemical and Physiological Functions in Very-Long-Chain Fatty Acid Elongation. Plant Physiol 167: 682–692

Haslam TM, Kunst L (2013) Extending the story of very-long-chain fatty acid elongation. Plant Sci 210: 93–107

Haslam TM, Mañas-Fernández A, Zhao L, Kunst L (2012) *Arabidopsis* ECERIFERUM2 is a component of the fatty acid elongation machinery required for fatty acid extension to exceptional lengths. Plant Physiol 160: 1164–1174

Hayashi H, De Bellis L, Ciurli A, Kondo M, Hayashi M, Nishimura M (1999) A novel acyl-CoA oxidase that can oxidize short-chain acyl-CoA in plant peroxisomes. J Biol Chem 274: 12715–12721

Heemskerk JW, Bögemann G, Scheijen MA, Wintermans JF (1986) Separation of chloroplast polar lipids and measurement of galactolipid metabolism by high-performance liquid chromatography. Anal Biochem 154: 85–91

Hernández ML, Whitehead L, He Z, Gazda V, Gilday A, Kozhevnikova E, Vaistij FE, Larson TR, Graham IA (2012) A cytosolic acyltransferase contributes to triacylglycerol synthesis in sucrose-rescued *Arabidopsis* seed oil catabolism mutants. Plant Physiol 160: 215–225

Hirano T, Sato MH (2011) Arabidopsis FAB1A/B is possibly involved in the recycling of auxin transporters. Plant Signal Behav 6: 583–585

Hobbs DH, Lu C, Hills MJ (1999) Cloning of a cDNA encoding diacylglycerol acyltransferase from *Arabidopsis thaliana* and its functional expression. FEBS Lett 452: 145–149

Hong Y, Devaiah SP, Bahn SC, Thamasandra BN, Li M, Welti R, Wang X (2009) Phospholipase D epsilon and phosphatidic acid enhance *Arabidopsis* nitrogen signaling and growth. Plant J 58: 376–387

Hooks MA, Kellas F, Graham IA (1999) Long-chain acyl-CoA oxidases of *Arabidopsis*. Plant J 20: 1–13

Hung CH, Kobayashi K, Wada H, Nakamura Y (2015) Isolation and characterization of a phosphatidylglycerophosphate phosphatase1, PGPP1, in *Chlamydomonas reinhardtii*. Plant Physiol Biochem 92: 56–61

Inatsugi R, Nakamura M, Nishida I (2002) Phosphatidylcholine biosynthesis at low temperature: differential expression of CTP:phosphorylcholine cytidylyltransferase isogenes in *Arabidopsis thaliana*. Plant Cell Physiol 43: 1342–1350

Ischebeck T, Stenzel I, Heilmann I (2008) Type B phosphatidylinositol-4-phosphate 5-kinases mediate *Arabidopsis* and *Nicotiana tabacum* pollen tube growth by regulating apical pectin secretion. Plant Cell 20: 3312–3330

Itoh R, Toda K, Takahashi H, Takano H, Kuroiwa T (1998) Delta-9 fatty acid desaturase gene containing a carboxyl-terminal cytochrome b5 domain from the red alga *Cyanidioschyzon merolae*. Curr Genet 33: 165–170

Jakab G, Manrique A, Zimmerli L, Métraux J-P, Mauch-Mani B (2003) Molecular characterization of a novel lipase-like pathogen-inducible gene family of *Arabidopsis*. Plant Physiol 132: 2230–2239

Joubès J, Raffaele S, Bourdenx B, Garcia C, Laroche-Traineau J, Moreau P, Domergue F, Lessire R (2008) The VLCFA elongase gene family in *Arabidopsis thaliana*: phylogenetic analysis, 3D modelling and expression profiling. Plant Mol Biol 67: 547–566

Joyard J, Ferro M, Masselon C, Seigneurin-Berny D, Salvi D, Garin J, Rolland N (2010) Chloroplast proteomics highlights the subcellular compartmentation of lipid metabolism. Prog Lipid Res 49: 128–158

Jung J, Kumar K, Lee HY, Park Y-I, Cho H-T, Ryu SB (2012) Translocation of phospholipase A2α to apoplasts is modulated by developmental stages and bacterial infection in *Arabidopsis*. Front Plant Sci 3: 126

Kachroo A, Shanklin J, Whittle E, Lapchyk L, Hildebrand D, Kachroo P (2007) The *Arabidopsis* stearoyl-acyl carrier protein-desaturase family and the contribution of leaf isoforms to oleic acid synthesis. Plant Mol Biol 63: 257–271

Katagiri T, Ishiyama K, Kato T, Tabata S, Kobayashi M, Shinozaki K (2005) An important role of phosphatidic acid in ABA signaling during germination in *Arabidopsis thaliana*. Plant J 43: 107–117

Katayama K, Sakurai I, Wada H (2004) Identification of an *Arabidopsis thaliana* gene for cardiolipin synthase located in mitochondria. FEBS Lett 577: 193–198

Kato T, Morita MT, Fukaki H, Yamauchi Y, Uehara M, Niihama M, Tasaka M (2002) SGR2, a phospholipase-like protein, and ZIG/SGR4, a SNARE, are involved in the shoot gravitropism of *Arabidopsis*. Plant Cell 14: 33–46

Kelly AA, Dörmann P (2002) *DGD2*, an arabidopsis gene encoding a UDP-galactose-dependent digalactosyldiacylglycerol synthase is expressed during growth under phosphate-limiting conditions. J Biol Chem 277: 1166–1173

Keogh MR, Courtney PD, Kinney AJ, Dewey RE (2009) Functional characterization of phospholipid *N*-methyltransferases from *Arabidopsis* and soybean. J Biol Chem 284: 15439–15447

Kim EY, Seo YS, Kim WT (2011a) AtDSEL, an *Arabidopsis* cytosolic DAD1-like acylhydrolase, is involved in negative regulation of storage oil mobilization during seedling establishment. J Plant Physiol 168: 1705–1709

Kim H, Li Y, Huang A (2005) Ubiquitous and endoplasmic reticulum–located lysophosphatidyl acyltransferase, LPAT2, is essential for female but not male gametophyte development in *Arabidopsis*. Plant Cell 17: 1073–1089

Kim HJ, Ok SH, Bahn SC, Jang J, Oh SA, Park SK, Twell D, Ryu SB, Shin JS (2011b) Endoplasmic Reticulum- and Golgi-Localized Phospholipase A2 Plays Critical Roles in *Arabidopsis* Pollen Development and Germination. Plant Cell 23: 94–110

Kim J, Jung JH, Lee SB, Go YS, Kim HJ, Cahoon R, Markham JE, Cahoon EB, Suh MC (2013) *Arabidopsis* 3-ketoacyl-coenzyme a synthase9 is involved in the synthesis of tetracosanoic acids as precursors of cuticular waxes, suberins, sphingolipids, and phospholipids. Plant Physiol 162: 567–580

Kobayashi K (2004) *Arabidopsis* Type B Monogalactosyldiacylglycerol Synthase Genes Are Expressed during Pollen Tube Growth and Induced by Phosphate Starvation. Plant Physiol 134: 640–648

Konishi T, Shinohara K, Yamada K, Sasaki Y (1996) Acetyl-CoA carboxylase in higher plants: most plants other than gramineae have both the prokaryotic and the eukaryotic forms of this enzyme. Plant Cell Physiol 37: 117–122

Kopka J, Ludewig M, Müller-Röber B (1997) Complementary DNAs encoding eukaryotic-type cytidine-5'-diphosphate-diacylglycerol synthases of two plant species. Plant Physiol 113: 997–1002

Lee Y, Kim Y-W, Jeon BW, Park K-Y, Suh SJ, Seo J, Kwak JM, Martinoia E, Hwang I, Lee Y (2007) Phosphatidylinositol 4,5-bisphosphate is important for stomatal opening. Plant J 52: 803–816

Li-Beisson Y, Beisson F, Riekhof W (2015) Metabolism of acyl-lipids in *Chlamydomonas reinhardtii*. Plant J 82: 504-522

Li M, Bahn SC, Guo L, Musgrave W, Berg H, Welti R, Wang X (2011) Patatin-related phospholipase pPLAIIIβ-induced changes in lipid metabolism alter cellulose content and cell elongation in *Arabidopsis*. Plant Cell 23: 1107–1123

Li X, Benning C, Kuo M-H (2012a) Rapid Triacylglycerol Turnover in *Chlamydomonas reinhardtii* Requires a Lipase with Broad Substrate Specificity. Eukaryot Cell 11: 1451–1462

Li X, Moellering ER, Liu B, Johnny C, Fedewa M, Sears BB, Kuo M-H, Benning C (2012b) A Galactoglycerolipid Lipase Is Required for Triacylglycerol Accumulation and Survival Following Nitrogen Deprivation in *Chlamydomonas reinhardtii*. Plant Cell 24: 4670–4686

Li Y, Beisson F, Koo AJK, Molina I, Pollard M, Ohlrogge J (2007) Identification of acyltransferases required for cutin biosynthesis and production of cutin with suberin-like monomers. Proc Natl Acad Sci U S A 104: 18339–18344

Lo M, Taylor C, Wang L, Nowack L (2004) Characterization of an ultraviolet B-induced lipase in *Arabidopsis*. Plant Physiol 135: 947–958

Löfke C, Ischebeck T, König S, Freitag S, Heilmann I (2008) Alternative metabolic fates of phosphatidylinositol produced by phosphatidylinositol synthase isoforms in *Arabidopsis thaliana*. Biochem J 413: 115–124

López-Marqués R, Poulsen LR, Hanisch S, Meffert K, Buch-Pedersen MJ, Jakobsen MK, Pomorski TG, Palmgren MG (2010) Intracellular targeting signals and lipid specificity determinants of the ALA/ALIS P4-ATPase complex reside in the catalytic ALA α-subunit. Mol Biol Cell 21: 791–801

López-Marqués RL, Poulsen LR, Palmgren MG (2012) A putative plant aminophospholipid flippase, the Arabidopsis P4 ATPase ALA1, localizes to the plasma membrane following association with a β-subunit. PLoS One 7: e33042

Lu B, Xu C, Awai K, Jones AD, Benning C (2007) A small ATPase protein of *Arabidopsis*, TGD3, involved in chloroplast lipid import. J Biol Chem 282: 35945–35953

Lu C, Xin Z, Ren Z, Miquel M (2009) An enzyme regulating triacylglycerol composition is encoded by the *ROD1* gene of *Arabidopsis*. Proc. Natl. Acad. Sci. U. S. A. 106: 18837–18842

McCartney A, Dyer J, Dhanoa P (2004) Membrane-bound fatty acid desaturases are inserted co-translationally into the ER and contain different ER retrieval motifs at their carboxy termini. Plant J 37: 156–173

Mikami K, Saavedra L, Hiwatashi Y, Uji T, Hasebe M, Sommarin M (2010) A dibasic amino acid pair conserved in the activation loop directs plasma membrane localization and is necessary for activity of plant type I/II phosphatidylinositol phosphate kinase. Plant Physiol 153: 1004–1015

Mizoi J, Nakamura M, Nishida I (2006) Defects in CTP:PHOSPHORYLETHANOLAMINE CYTIDYLYLTRANSFERASE affect embryonic and postembryonic development in *Arabidopsis*. Plant Cell 18: 3370–3385

Moellering ER, Muthan B, Benning C (2010) Freezing tolerance in plants requires lipid remodeling at the outer chloroplast membrane. Science 330: 226–228

Morita MT, Kato T, Nagafusa K, Saito C, Ueda T, Nakano A, Tasaka M (2002) Involvement of the vacuoles of the endodermis in the early process of shoot gravitropism in *Arabidopsis*. Plant Cell 14: 47–56

Müller F, Frentzen M (2001) Phosphatidylglycerophosphate synthases from *Arabidopsis thaliana*. FEBS Lett 509: 298–302

Nakamura Y, Awai K, Masuda T, Yoshioka Y, Takamiya K, Ohta H (2005) A novel phosphatidylcholine-hydrolyzing phospholipase C induced by phosphate starvation in *Arabidopsis*. J Biol Chem 280: 7469–7476

Nakamura Y, Koizumi R, Shui G, Shimojima M, Wenk MR, Ito T, Ohta H (2009) *Arabidopsis* lipins mediate eukaryotic pathway of lipid metabolism and cope critically with phosphate starvation. Proc Natl Acad Sci U S A 106: 20978–20983

Nakamura Y, Tsuchiya M, Ohta H (2007) Plastidic phosphatidic acid phosphatases identified in a distinct subfamily of lipid phosphate phosphatases with prokaryotic origin. J Biol Chem 282: 29013–29021

Naoki S, Norio M (1982) Lipid biosynthesis in the blue-green alga, *Anabaena variabilis*. Biochim Biophys Acta - Lipids Lipid Metab 710: 271–278

Nerlich A, von Orlow M, Rontein D, Hanson AD, Dörmann P (2007) Deficiency in phosphatidylserine decarboxylase activity in the *psd1 psd2 psd3* triple mutant of *Arabidopsis* affects phosphatidylethanolamine accumulation in mitochondria. Plant Physiol 144: 904–914

Nishida I, Murata N (1996) CHILLING SENSITIVITY IN PLANTS AND CYANOBACTERIA: The Crucial Contribution of Membrane Lipids. Annu Rev Plant Physiol Plant Mol Biol 47: 541–568

Nishida I, Tasaka Y, Shiraishi H, Murata N (1993) The gene and the RNA for the precursor to the plastid-located glycerol-3-phosphate acyltransferase of *Arabidopsis thaliana*. Plant Mol Biol 21: 267–277

Nowicki M, Müller F, Frentzen M (2005) Cardiolipin synthase of *Arabidopsis thaliana*. FEBS Lett 579: 2161–2165

Nuccio ML, Ziemak MJ, Henry SA, Weretilnyk EA, Hanson AD (2000) cDNA cloning of phosphoethanolamine *N*-methyltransferase from spinach by complementation in *Schizosaccharomyces pombe* and characterization of the recombinant enzyme. J Biol Chem 275: 14095–14101

Okazaki K, Sato N, Tsuji N, Tsuzuki M, Nishida I (2006) The significance of C16 fatty acids in the *sn*-2 positions of glycerolipids in the photosynthetic growth of *Synechocystis* sp. PCC6803. Plant Physiol 141: 546–556

Okuley J (1994) *Arabidopsis* FAD2 Gene Encodes the Enzyme That Is Essential for Polyunsaturated Lipid Synthesis. Plant Cell 6: 147–158

Pascal S, Bernard A, Sorel M, Pervent M, Vile D, Haslam RP, Napier JA, Lessire R, Domergue F, Joubès J (2013) The *Arabidopsis cer26* mutant, like the *cer2* mutant, is specifically affected in the very long chain fatty acid elongation process. Plant J 73: 733–746

Pidkowich MS, Nguyen HT, Heilmann I, Ischebeck T, Shanklin J (2007) Modulating seed beta-ketoacyl-acyl carrier protein synthase II level converts the composition of a temperate seed oil to that of a palm-like tropical oil. Proc Natl Acad Sci U S A 104: 4742–4747

Pierrugues O, Brutesco C, Oshiro J, Gouy M, Deveaux Y, Carman GM, Thuriaux P, Kazmaier M (2001) Lipid phosphate phosphatases in *Arabidopsis*. Regulation of the *AtLPP1* gene in response to stress. J Biol Chem 276: 20300–20308

Pokotylo I, Pejchar P, Potocký M, Kocourková D, Krčková Z, Ruelland E, Kravets V, Martinec J (2013) The plant non-specific phospholipase C gene family. Novel competitors in lipid signalling. Prog Lipid Res 52: 62–79

Poulsen LR, Lopez-Marques RL, McDowell SC, Okkeri J, Licht D, Schulz A, Pomorski T, Harper JF, Palmgren MG (2008) The Arabidopsis P4-ATPase ALA3 Localizes to the Golgi and Requires a β-Subunit to Function in Lipid Translocation and Secretory Vesicle Formation. Plant Cell 20: 658–676

Pulsifer IP, Lowe C, Narayaran SA, Busuttil AS, Vishwanath SJ, Domergue F, Rowland O (2014) Acyl-lipid thioesterase1-4 from *Arabidopsis thaliana* form a novel family of fatty acyl-acyl carrier protein thioesterases with divergent expression patterns and substrate specificities. Plant Mol Biol 84: 549–563

Puyaubert J, Denis L, Alban C (2008) Dual targeting of *Arabidopsis* holocarboxylase synthetase1: a small upstream open reading frame regulates translation initiation and protein targeting. Plant Physiol 146: 478–491

Quist TM, Sokolchik I, Shi H, Joly RJ, Bressan RA, Maggio A, Narsimhan M, Li X (2009) *HOS3*, an ELO-like gene, inhibits effects of ABA and implicates a S-1-P/ceramide control system for abiotic stress responses in *Arabidopsis thaliana*. Mol Plant 2: 138–151

Richmond TA, Bleecker AB (1999) A defect in beta-oxidation causes abnormal inflorescence development in *Arabidopsis*. Plant Cell 11: 1911–1924

Riekhof WR, Andre C, Benning C (2005) Two enzymes, BtaA and BtaB, are sufficient for betaine lipid biosynthesis in bacteria. Arch Biochem Biophys 441: 96–105

Rylott EL, Eastmond PJ, Gilday AD, Slocombe SP, Larson TR, Baker A, Graham IA (2006) The *Arabidopsis thaliana* multifunctional protein gene (MFP2) of peroxisomal beta-oxidation is essential for seedling establishment. Plant J 45: 930–941

Rylott EL, Rogers CA, Gilday AD, Edgell T, Larson TR, Graham IA (2003) *Arabidopsis* mutants in short- and medium-chain acyl-CoA oxidase activities accumulate acyl-CoAs and reveal that fatty acid beta-oxidation is essential for embryo development. J Biol Chem 278: 21370–21377

Sakurai I, Mizusawa N, Wada H, Sato N (2007) Digalactosyldiacylglycerol is required for stabilization of the oxygen-evolving complex in photosystem II. Plant Physiol 145: 1361–1370

Salas JJ, Ohlrogge JB (2002) Characterization of substrate specificity of plant FatA and FatB acyl-ACP thioesterases. Arch Biochem Biophys 403: 25–34

Sasaki Y, Nagano Y (2004) Plant acetyl-CoA carboxylase: structure, biosynthesis, regulation, and gene manipulation for plant breeding. Biosci Biotechnol Biochem 68: 1175–1184

Sato N (2009) Gclust: trans-kingdom classification of proteins using automatic individual threshold setting. Bioinformatics 25: 599–605

Sato N, Moriyama T (2007) Genomic and biochemical analysis of lipid biosynthesis in the unicellular rhodophyte *Cyanidioschyzon merolae*: lack of a plastidic desaturation pathway results in the coupled pathway of galactolipid synthesis. Eukaryot Cell 6: 1006–1017

Seo J, Lee HY, Choi H, Choi Y, Lee Y, Kim Y-W, Ryu SB, Lee Y (2008) Phospholipase A2β mediates light-induced stomatal opening in *Arabidopsis*. J Exp Bot 59: 3587–3594

Seo YS, Kim EY, Kim JH, Kim WT (2009) Enzymatic characterization of class I DAD1-like acylhydrolase members targeted to chloroplast in *Arabidopsis*. FEBS Lett 583: 2301–2307

Seo YS, Kim EY, Kim WT (2011) The Arabidopsis *sn*-1-specific mitochondrial acylhydrolase AtDLAH is positively correlated with seed viability. J Exp Bot 62: 5683–5698

Shimada H (2004) ARC3, a Chloroplast Division Factor, is a Chimera of Prokaryotic FtsZ and Part of Eukaryotic Phosphatidylinositol-4-phosphate 5-kinase. Plant Cell Physiol 45: 960–967

Shimakata T, Stumpf PK (1982) Isolation and function of spinach leaf beta-ketoacyl-[acyl-carrier-protein] synthases. Proc Natl Acad Sci U S A 79: 5808–5812

Shintani DK, Ohlrogge JB (1994) The characterization of a mitochondrial acyl carrier protein isoform isolated from *Arabidopsis thaliana*. Plant Physiol 104: 1221–1229

Smith MA, Dauk M, Ramadan H, Yang H, Seamons LE, Haslam RP, Beaudoin F, Ramirez-Erosa I, Forseille L (2013) Involvement of Arabidopsis ACYL-COENZYME A DESATURASE-LIKE2 (At2g31360) in the biosynthesis of the very-long-chain monounsaturated fatty acid components of membrane lipids. Plant Physiol 161: 81–96

Ståhl U, Carlsson AS, Lenman M, Dahlqvist A, Huang B, Banas W, Banas A, Stymne S (2004) Cloning and functional characterization of a phospholipid:diacylglycerol acyltransferase from *Arabidopsis*. Plant Physiol 135: 1324–1335

Ståhl U, Stålberg K, Stymne S, Ronne H (2008) A family of eukaryotic lysophospholipid acyltransferases with broad specificity. FEBS Lett 582: 305–309

Stålberg K, Ståhl U, Stymne S, Ohlrogge J (2009) Characterization of two *Arabidopsis thaliana* acyltransferases with preference for lysophosphatidylethanolamine. BMC Plant Biol 9: 60

Stevenson-Paulik J, Love J, Boss WF (2003) Differential regulation of two *Arabidopsis* type III phosphatidylinositol 4-kinase isoforms. A regulatory role for the pleckstrin homology domain. Plant Physiol 132: 1053–1064

Tanoue R, Kobayashi M, Katayama K, Nagata N, Wada H (2014) Phosphatidylglycerol biosynthesis is required for the development of embryos and normal membrane structures of chloroplasts and mitochondria in *Arabidopsis*. FEBS Lett 588: 1680–1685

Tasseva G, Richard L, Zachowski A (2004) Regulation of phosphatidylcholine biosynthesis under salt stress involves choline kinases in *Arabidopsis thaliana*. FEBS Lett 566: 115–120

Van Besouw A, Wintermans JF (1978) Galactolipid formation in chloroplast envelopes. I. Evidence for two mechanisms in galactosylation. Biochim Biophys Acta 529: 44–53

Wang Z, Anderson NS, Benning C (2013) The phosphatidic acid binding site of the *Arabidopsis* trigalactosyldiacylglycerol 4 (TGD4) protein required for lipid import into chloroplasts. J Biol Chem 288: 4763–4771

Wang Z, Xu C, Benning C (2012) TGD4 involved in endoplasmic reticulum-to-chloroplast lipid trafficking is a phosphatidic acid binding protein. Plant J 70: 614–623

Weier D, Müller C, Gaspers C, Frentzen M (2005) Characterisation of acyltransferases from *Synechocystis* sp. PCC6803. Biochem Biophys Res Commun 334: 1127–1134

Weng H, Molina I, Shockey J, Browse J (2010) Organ fusion and defective cuticle function in a *lacs1 lacs2* double mutant of Arabidopsis. Planta 231: 1089–1100

Willige B, Ghosh S, Nill C (2007) The DELLA domain of GA INSENSITIVE mediates the interaction with the GA INSENSITIVE DWARF1A gibberellin receptor of *Arabidopsis*. Plant Cell 19: 1209–1220

Xiao S, Chye M-L (2011) New roles for acyl-CoA-binding proteins (ACBPs) in plant development, stress responses and lipid metabolism. Prog Lipid Res 50: 141–151

Xu C, Fan J, Froehlich JE, Awai K, Benning C (2005) Mutation of the TGD1 chloroplast envelope protein affects phosphatidate metabolism in *Arabidopsis*. Plant Cell 17: 3094–3110

Xu C, Härtel H, Wada H, Hagio M, Yu B, Eakin C, Benning C (2002) The *pgp1* mutant locus of Arabidopsis encodes a phosphatidylglycerolphosphate synthase with impaired activity. Plant Physiol 129: 594–604

Xu C, Yu B, Cornish AJ, Froehlich JE, Benning C (2006) Phosphatidylglycerol biosynthesis in chloroplasts of *Arabidopsis* mutants deficient in acyl-ACP glycerol-3-phosphate acyltransferase. Plant J 47: 296–309

Xue HW, Hosaka K, Plesch G, Mueller-Roeber B (2000) Cloning of *Arabidopsis thaliana* phosphatidylinositol synthase and functional expression in the yeast *pis* mutant. Plant Mol Biol 42: 757–764

Yamaoka Y, Yu Y, Mizoi J, Fujiki Y, Saito K, Nishijima M, Lee Y, Nishida I (2011) PHOSPHATIDYLSERINE SYNTHASE1 is required for microspore development in *Arabidopsis thaliana.* Plant J 67: 648–661

Yamaryo Y, Dubots E, Albrieux C, Baldan B, Block MA (2008) Phosphate availability affects the tonoplast localization of PLDζ2, an *Arabidopsis thaliana* phospholipase D. FEBS Lett 582: 685–690

Yang W, Pollard M, Li-Beisson Y, Beisson F, Feig M, Ohlrogge J (2010) A distinct type of glycerol-3-phosphate acyltransferase with *sn*-2 preference and phosphatase activity producing 2-monoacylglycerol. Proc Natl Acad Sci U S A 107: 12040–12045

Yang W, Simpson JP, Li-Beisson Y, Beisson F, Pollard M, Ohlrogge JB (2012) A land-plant-specific glycerol-3-phosphate acyltransferase family in *Arabidopsis*: substrate specificity, *sn*-2 preference, and evolution. Plant Physiol 160: 638–652

Yasuno R, von Wettstein-Knowles P, Wada H (2004) Identification and molecular characterization of the beta-ketoacyl-[acyl carrier protein] synthase component of the *Arabidopsis* mitochondrial fatty acid synthase. J Biol Chem 279: 8242–8251

Yu B, Wakao S, Fan J, Benning C (2004) Loss of plastidic lysophosphatidic acid acyltransferase causes embryo-lethality in *Arabidopsi*s. Plant Cell Physiol 45: 503–510

Yu B, Xu C, Benning C (2002) *Arabidopsis* disrupted in *SQD2* encoding sulfolipid synthase is impaired in phosphate-limited growth. Proc Natl Acad Sci U S A 99: 5732–5737

Zhang M, Fan J, Taylor DC, Ohlrogge JB (2009) DGAT1 and PDAT1 acyltransferases have overlapping functions in *Arabidopsis* triacylglycerol biosynthesis and are essential for normal pollen and seed development. Plant Cell 21: 3885–3901

Zhao L, Katavic V, Li F, Haughn GW, Kunst L (2010a) Insertional mutant analysis reveals that long-chain acyl-CoA synthetase 1 (LACS1), but not LACS8, functionally overlaps with LACS9 in *Arabidopsis* seed oil biosynthesis. Plant J 64: 1048–1058

Zhao Y, Yan A, Feijo JA, Furutani M, Takenawa T, Hwang I, Fu Y, Yang Z (2010b) Phosphoinositides Regulate Clathrin-Dependent Endocytosis at the Tip of Pollen Tubes in *Arabidopsis* and Tobacco. Plant Cell 22: 4031–4044

Zheng H, Rowland O, Kunst L (2005) Disruptions of the *Arabidopsis* Enoyl-CoA reductase gene reveal an essential role for very-long-chain fatty acid synthesis in cell expansion during plant morphogenesis. Plant Cell 17: 1467–1481

Zheng S-Z, Liu Y-L, Li B, Shang Z, Zhou R-G, Sun D-Y (2012) Phosphoinositide-specific phospholipase C9 is involved in the thermotolerance of *Arabidopsis*. Plant J 69: 689–700

Zheng Z, Xia Q, Dauk M, Shen W (2003) *Arabidopsis AtGPAT1*, a member of the membrane-bound glycerol-3-phosphate acyltransferase gene family, is essential for tapetum differentiation and male fertility. Plant Cell 15: 1872–1887

Zhou Y, Peisker H, Weth A, Baumgartner W, Dörmann P, Frentzen M (2013) Extraplastidial cytidinediphosphate diacylglycerol synthase activity is required for vegetative development in *Arabidopsis thaliana*. Plant J 75: 867–879

Zolman BK, Nyberg M, Bartel B (2007) IBR3, a novel peroxisomal acyl-CoA dehydrogenase-like protein required for indole-3-butyric acid response. Plant Mol Biol 64: 59–72

Zou J, Wei Y, Jako C, Kumar A, Selvaraj G, Taylor DC (1999) The *Arabidopsis thaliana TAG1* mutant has a mutation in a diacylglycerol acyltransferase gene. Plant J 19: 645–653

2 Supplementary Figures and Tables

2.1 Supplementary Figures

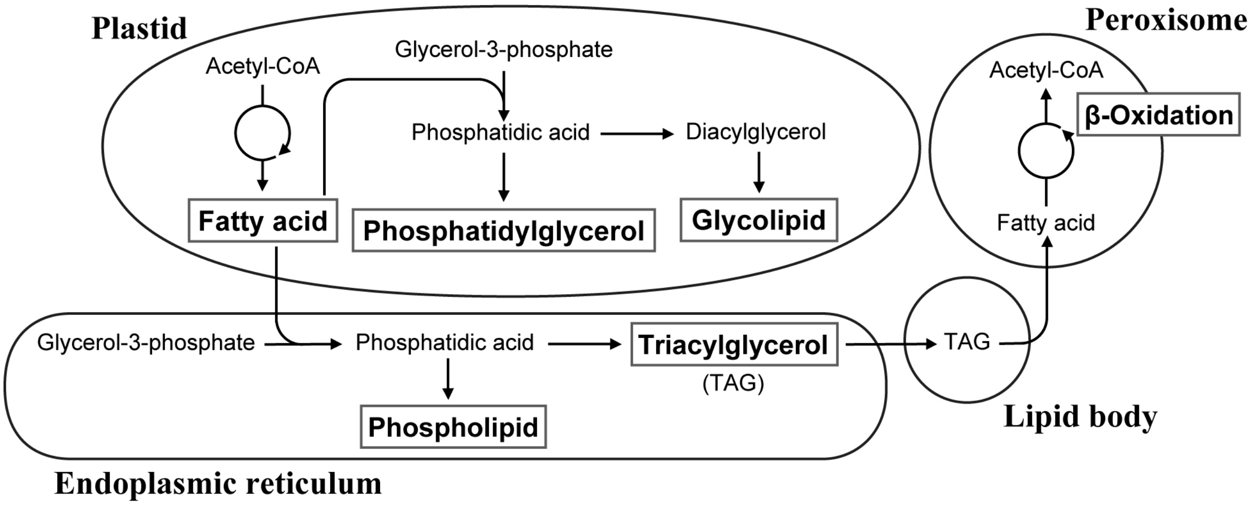

Supplementary Figure 1. A schematic diagram of lipid metabolism in plants (Li-Beisson et al., 2013).

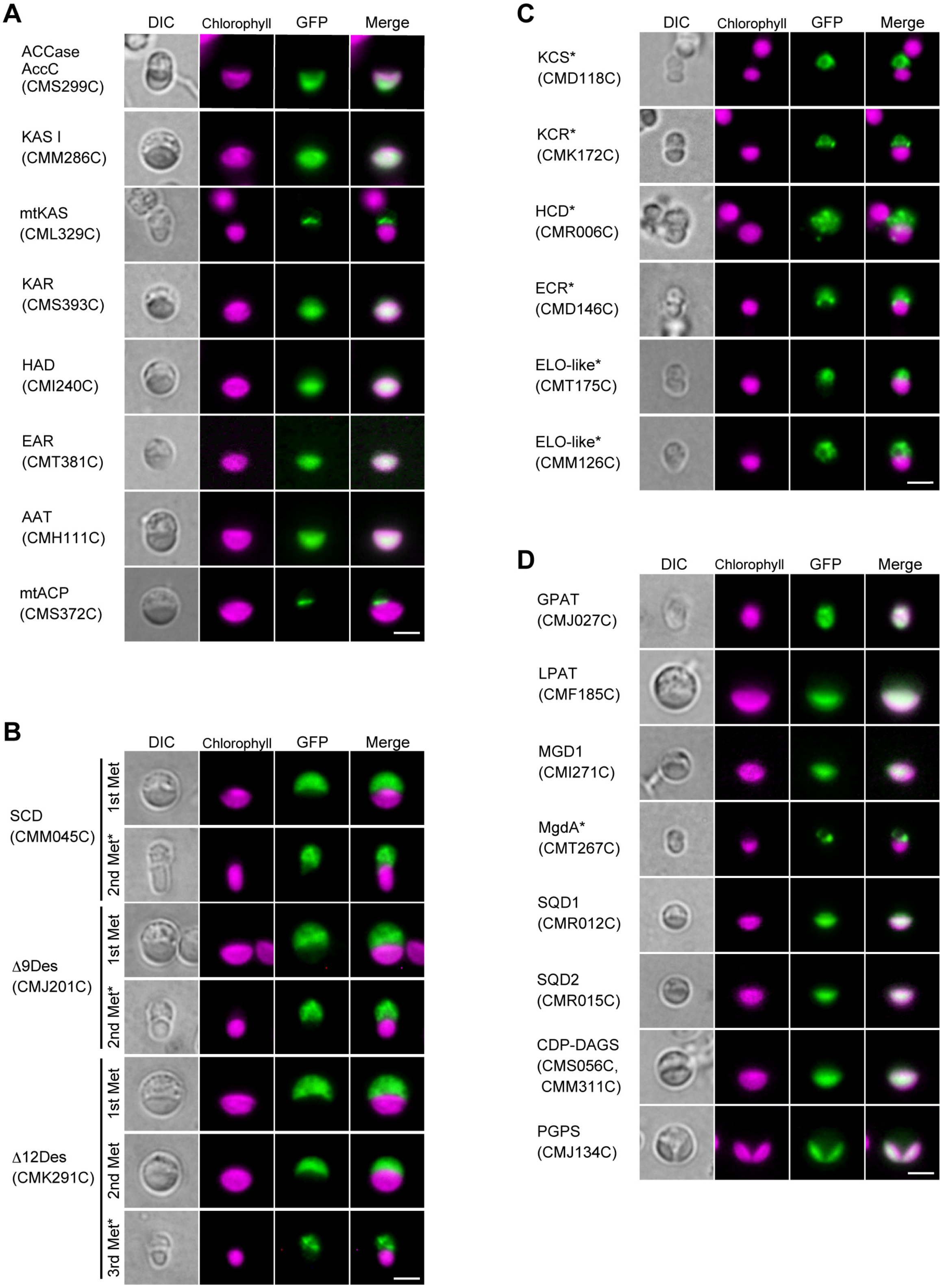

Supplementary Figure 2. Subcellular localization of enzymes related to fatty acid synthesis, fatty acid elongation, desaturation and plastidic lipid synthesis in *C. merolae*. These fluorescence micrographs show *C. merolae* cells transiently expressing GFP- or HA-fused protein related to fatty

acid synthesis (A), desaturation (B), fatty acid elongation (C), and synthesis of glycolipids and PG (D). Abbreviation of enzyme names is indicated to Table 1. Subcellular localization of CMM311C was not analyzed, because amino acid sequence of this protein is identical to CMS056C. GPAT (CMJ027C) and mtKAS (CML329C) were examined subcellular localization using HA-tag construct. Asterisked enzymes of subcellular localization were detected by immunostained with anti-GFP antibody. DIC; Nomarski differential interference contrast, Chlorophyll; phycobilin and chlorophyll autofluorescences, GFP; GFP fluorescence or immunofluorescence using anti-GFP or anti-HA tag antibody, Merge; merged images of phycobilin and chlorophyll autofluorescences as well as green fluorescence. Bar = 2 μm.

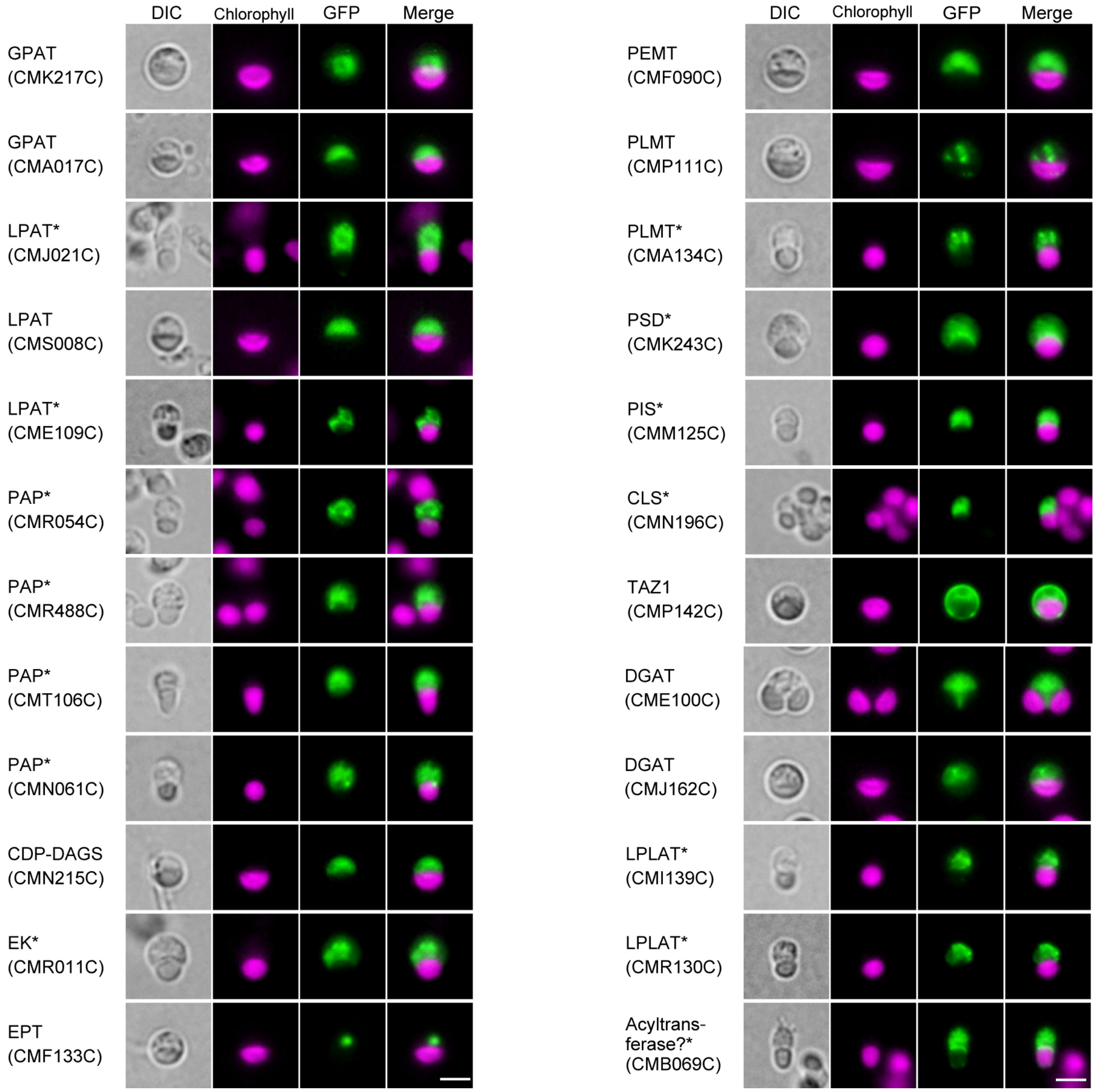

Supplementary Figure 3. Subcellular localization of enzymes related to synthesis of phospholipids and TAG in *C. merolae*. These fluorescence micrographs show *C. merolae* cells transiently expressing GFP-fused protein related to synthesis of phospholipids and TAG. Abbreviation of enzyme names is indicated to Table 1. Asterisked enzymes of subcellular localization were detected by immunostained with anti-GFP antibody. DIC; Nomarski differential interference contrast, Chlorophyll; phycobilin and chlorophyll autofluorescences, GFP; GFP fluorescence or immunofluorescence using anti-GFP antibody, Merge; merged images of phycobilin and chlorophyll autofluorescences as well as green fluorescence. Bar = 2 μm.

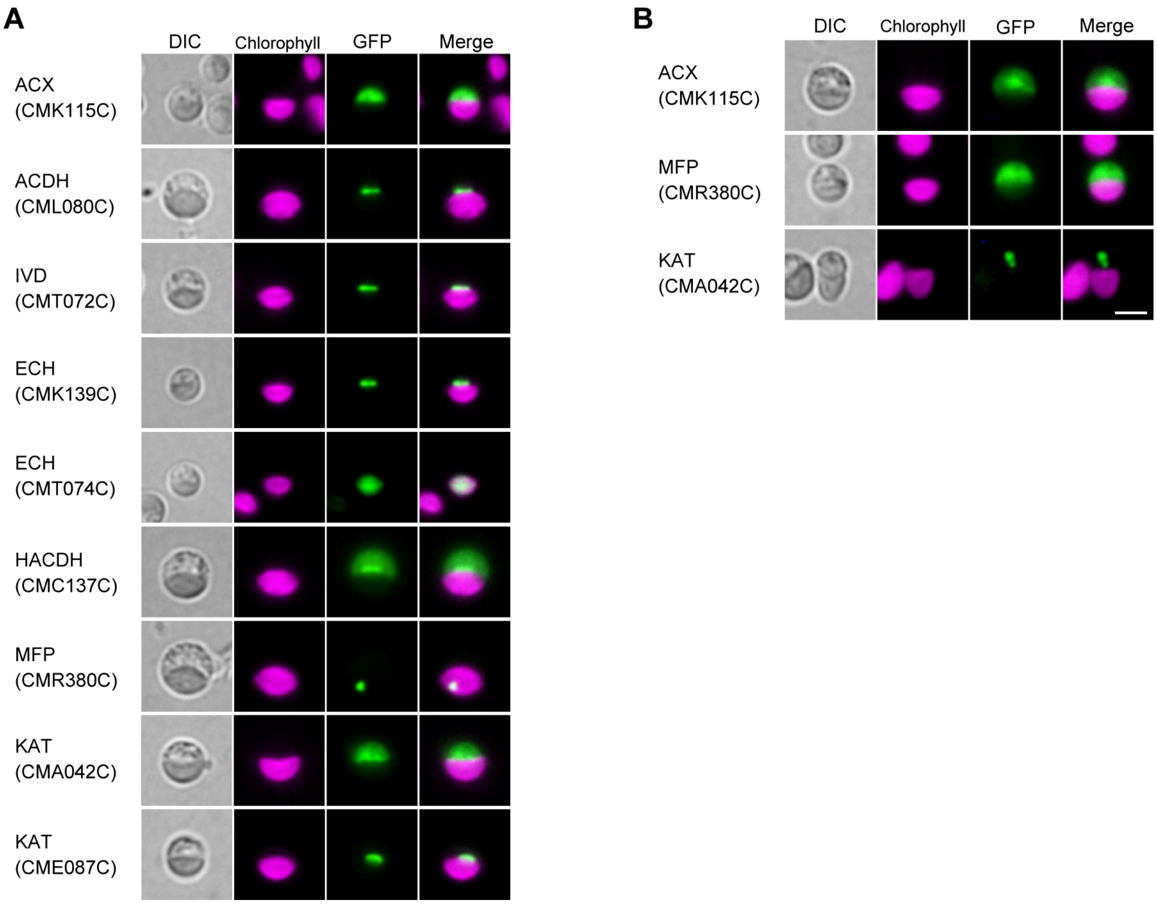

Supplementary Figure 4. Subcellular localization of enzymes related to β-oxidation. Fluorescence micrographs show *C. merolae* cells transiently expressing GFP-fused protein related to β-oxidation (A). Three β-oxidation enzymes were examined subcellular localization using constructs of GFP-fused *C*-terminal peptide (B). Abbreviation of enzyme names is indicated to Table 1. DIC; Nomarski differential interference contrast, Chlorophyll; phycobilin and chlorophyll autofluorescences, GFP; GFP fluorescence, Merge; merged images of phycobilin and chlorophyll autofluorescences as well as green fluorescence. Bar = 2 µm.

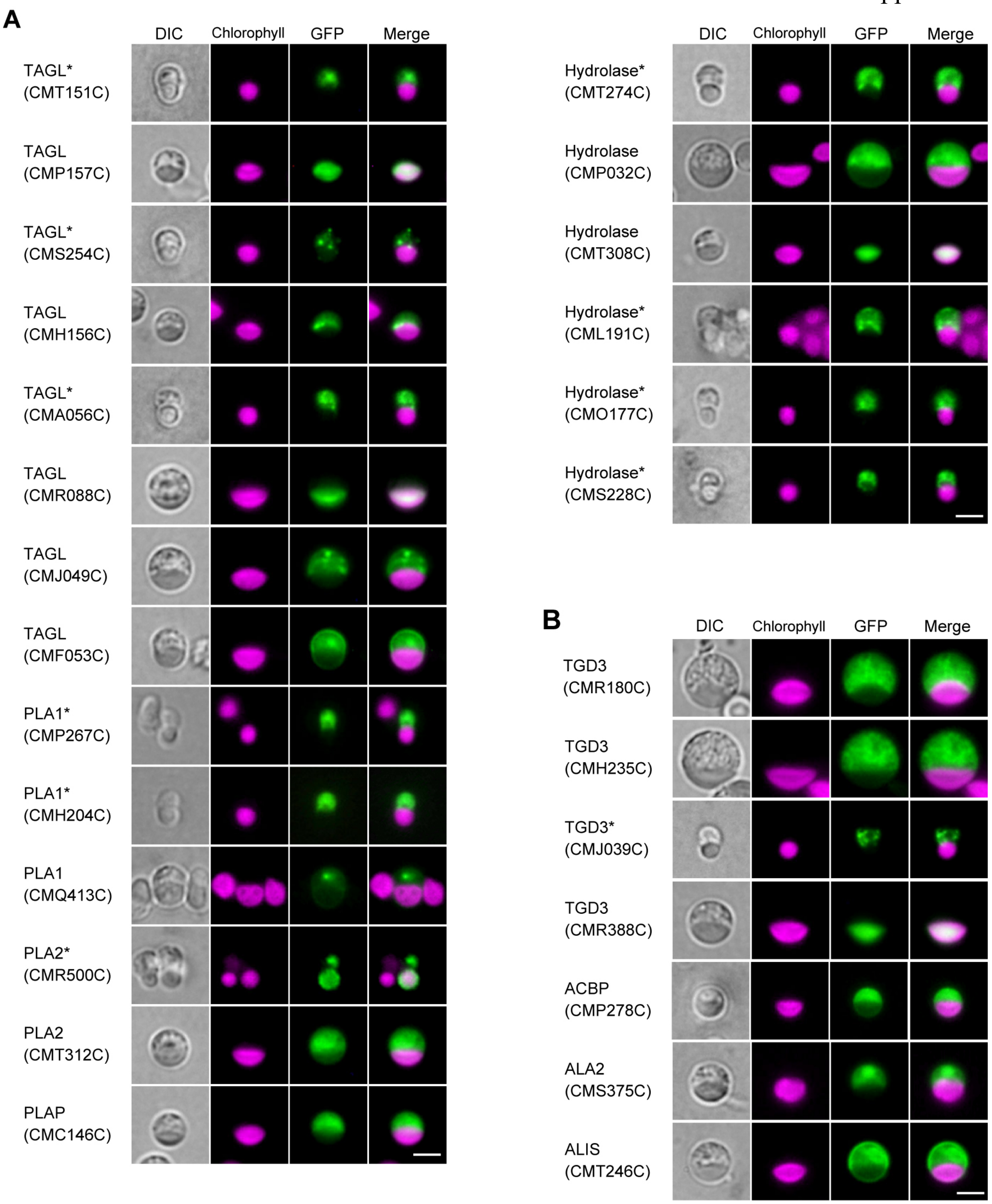

Supplementary Figure 5. Subcellular localization of enzymes related to lipid degradation, lipid trafficking in *C. merolae*. Fluorescence micrographs show *C. merolae* cells transiently expressing GFP-fused protein related to lipid degradation (A) and lipid trafficking (B). Abbreviation of enzyme names is indicated to Supplementary Table 3. Asterisked enzymes of subcellular localization were detected by immunostained with anti-GFP antibody. DIC; Nomarski differential interference contrast, Chlorophyll; phycobilin and chlorophyll autofluorescences, GFP; GFP fluorescence or immunofluorescence using anti-GFP antibody, Merge; merged images of phycobilin and chlorophyll autofluorescences as well as green fluorescence. Bar = 2 µm.

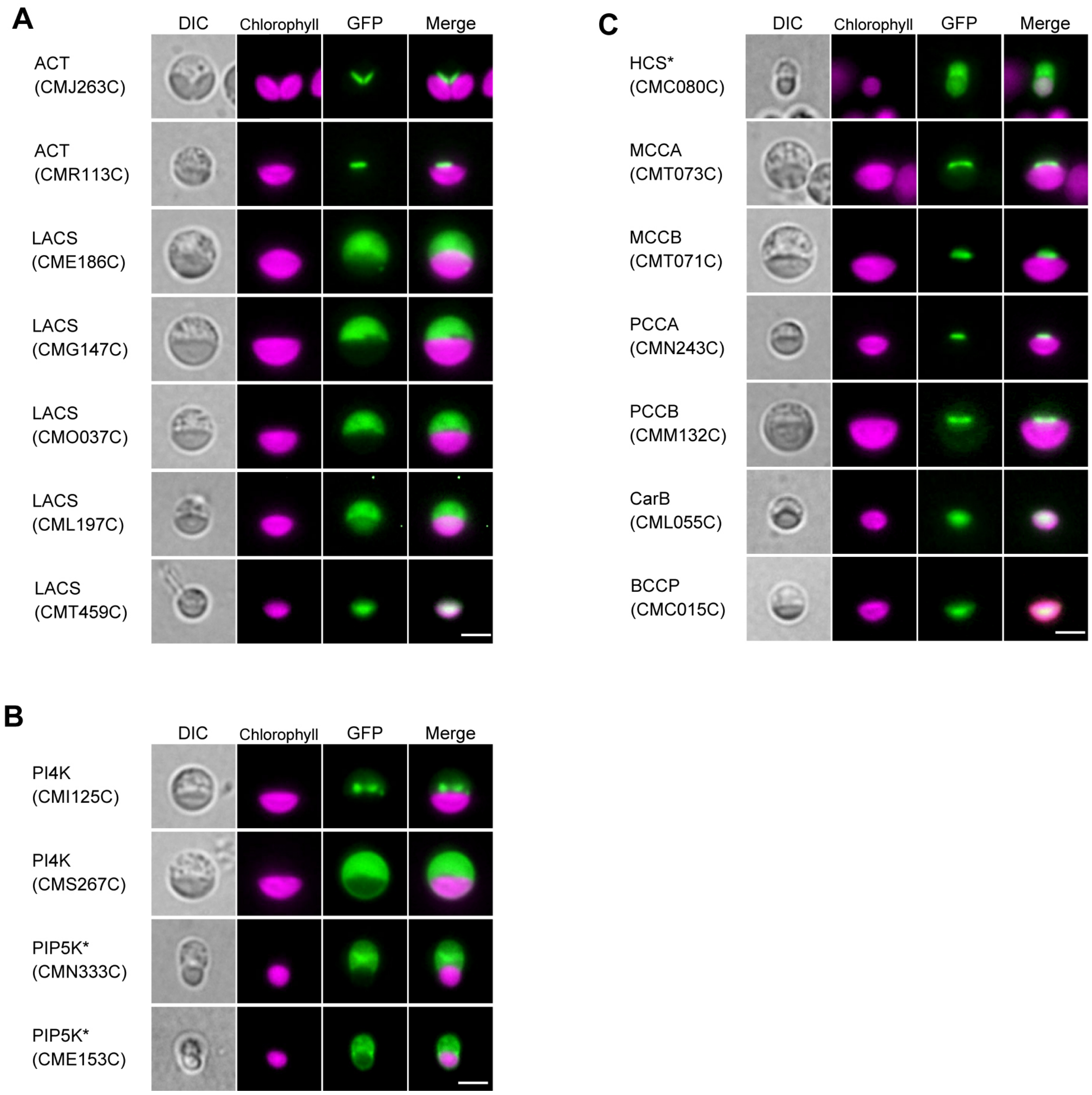

Supplementary Figure 6. Subcellular localization of enzymes related to fatty acid activation, PI signaling and biotin-dependent carboxylation in *C. merolae*. These fluorescence micrographs show *C. merolae* cells transiently expressing GFP-fused protein related to fatty acid activation (A), PI signaling (B) and biotin-dependent carboxylation (C). Abbreviation of enzyme names is indicated to Supplementary Table 3. Asterisked enzymes of subcellular localization were detected by immunostained with anti-GFP antibody. DIC; Nomarski differential interference contrast, Chlorophyll; phycobilin and chlorophyll autofluorescences, GFP; GFP fluorescence or immunofluorescence using anti-GFP antibody, Merge; merged images of phycobilin and chlorophyll autofluorescences as well as green fluorescence. Bar = 2 μm.

2.2 Supplementary Tables

Supplementary Table 1. List of primers used for making of EGFP or HA tag constructs.

A part of uppercase letters of sequence of primers indicates common sequences of pCEG1 or pBSHAb-T3´ vector required for the cloning using the In-Fusion Cloning Kit (Clontech laboratories, Mountain View, CA, USA). Because a peptide sequence of CMS056C and CMM311C is identical, the same primer is used for subcellular localization analysis of these proteins.

Locus tag	Sequence of forward primer (5´ to 3´)	Sequence of reverse primer (5´ to 3´)	Cloned length (aa)	Full length of enzyme (aa)
***N*-terminal peptide-EGFP construct**				
CMK217C	TTCGTTGACCTCTAGAatgctttttgtacgcaactg	CCATGGATCCTCTAGAgtgcagccgcgagagtctcc	103	673
CMA017C	TTCGTTGACCTCTAGAatggcagcgaccaccgccaa	CCATGGATCCTCTAGAcgtagataactcctgggtca	110	556
CMJ021C	TTCGTTGACCTCTAGAatgatccgtgatccataccg	CCATGGATCCTCTAGAggcacgctgatgaataccag	32	202
CMF185C	TTCGTTGACCTCTAGAatgtacctagctttggggtc	CCATGGATCCTCTAGAgcgagtgtcgagcctgcagc	91	373
CMS008C	TTCGTTGACCTCTAGAatggaaacagcacctctact	CCATGGATCCTCTAGAaaacgggttccgcaccagtt	103	430
CMR054C	TTCGTTGACCTCTAGAatggaacgggaaccaggtgc	CCATGGATCCTCTAGAcgggtaccaatgattcgcgt	69	294
CMR488C	TTCGTTGACCTCTAGAatgaacggcagttacgctct	CCATGGATCCTCTAGAcggaagttgaacgctgggat	60	302
CMT106C	TTCGTTGACCTCTAGAatggttcctcagcagacgcc	CCATGGATCCTCTAGAgccggccccgcacaagacga	74	363
CMT239C	TTCGTTGACCTCTAGAatgcttctggcggaaagcct	CCATGGATCCTCTAGAgcaccaggtatttcgcctct	142	532
CMN061C	TTCGTTGACCTCTAGAatgcatacactgcatgaaga	CCATGGATCCTCTAGAtcgggcgtatacgctactgg	75	944
CMT267C	TTCGTTGACCTCTAGAatgttgccggaaacgagcaa	CCATGGATCCTCTAGAgaacgggagacttccagtat	73	450
CMI271C	TTCGTTGACCTCTAGAatgcgcacccttccggcttt	CCATGGATCCTCTAGAactcgggtaaacagaggcta	66	683
CMR012C	TTCGTTGACCTCTAGAatgacgttcgtgacgctacc	CCATGGATCCTCTAGAcccaagaacaagaacagttt	80	511
CMR015C	TTCGTTGACCTCTAGAatgttcactggtcgaacagc	CCATGGATCCTCTAGAcactcgacgccgaggcgcgc	100	517
CMN215C	TTCGTTGACCTCTAGAatgcgcagcggaggaccgga	CCATGGATCCTCTAGAcgtgctttcgccagcttccc	90	542
CMS056C, CMM311C	TTCGTTGACCTCTAGAatgtgggtgtctacagtgca	CCATGGATCCTCTAGAtcggacagccagatcacgtg	133	439
CMJ134C	TTCGTTGACCTCTAGAatgcgtcagagcatcgaatc	CCATGGATCCTCTAGAattgggcaagttccaaaagc	248	429
CMN196C	TTCGTTGACCTCTAGAatgcacgccgcaaacctggt	CCATGGATCCTCTAGAggccaaggcagctatactat	33	208
CMM125C	TTCGTTGACCTCTAGAatgacaggaaaggcagcttg	CCATGGATCCTCTAGAcggtacgttccagaaggcga	33	237
CMR011C	TTCGTTGACCTCTAGAatggtgctgcaaacccttga	CCATGGATCCTCTAGAgccaaagattcgaacgagaa	84	385
CMS052C	TTCGTTGACCTCTAGAatggataacaaccccggtcg	CCATGGATCCTCTAGAaccaacaatgagttcgtccc	92	442

CMF133C	TTCGTTGACCTCTAGAatgtcacctgcgttgtctaa	CCATGGATCCTCTAGAaagctcttcagttgacaaat	78	554
CMF090C	TTCGTTGACCTCTAGAatggagttaccgagcgtcca	CCATGGATCCTCTAGAcggcgacgctggcggtacac	102	749
CMP111C	TTCGTTGACCTCTAGAatggatacgttaggagctcg	CCATGGATCCTCTAGAcggtgctgcgatgatgtacg	66	264
CMA134C	TTCGTTGACCTCTAGAatgttggcacttgatcgctg	CCATGGATCCTCTAGAcggcgctaaaatgatgctga	99	311
CMQ199C	TTCGTTGACCTCTAGAatgcgtgcttgtgtgcgctg	CCATGGATCCTCTAGAcgctgggcaacgaaacaaga	130	572
CME100C	TTCGTTGACCTCTAGAatgcgtcgacgtcagaactc	CCATGGATCCTCTAGAatctttctccgggagacgag	118	506
CMJ162C	TTCGTTGACCTCTAGAatgcctctacggacctacat	CCATGGATCCTCTAGAttgactcgaatctaggatgc	88	310
CMI139C	TTCGTTGACCTCTAGAatgaatgcgctgtctgccca	CCATGGATCCTCTAGAccatctagccgatggcagac	82	550
CMK243C	TTCGTTGACCTCTAGAatggtccgtcaacgaacgct	CCATGGATCCTCTAGAatctagctgacaggcaggta	92	384
CMR180C	TTCGTTGACCTCTAGAatgaacggtacacgagtctt	CCATGGATCCTCTAGAgaactgggcgaataattgtc	65	636
CMJ039C	TTCGTTGACCTCTAGAatgggactggtctcttttcg	CCATGGATCCTCTAGAccccgtcgttcgagagaaaa	67	1055
CMH235C	TTCGTTGACCTCTAGAatggaggcgggtataggttc	CCATGGATCCTCTAGAatcgttactaaacaccttga	233	996
CMR388C	TTCGTTGACCTCTAGAatgtggcagactgggctgca	CCATGGATCCTCTAGActtgtatacgtcctctactc	93	455
CMR306C	TTCGTTGACCTCTAGAatgaggttcgaggcatcggc	CCATGGATCCTCTAGAgttgaacgtcgggttattga	92	1334
CMS375C	TTCGTTGACCTCTAGAatggacccacagcgacgtac	CCATGGATCCTCTAGActccaggtatatggtgcgtg	168	1157
CMT246C	TTCGTTGACCTCTAGAatggtcactgtggaggacct	CCATGGATCCTCTAGAgaagagcggtttcaccgctg	210	527
CMI125C	TTCGTTGACCTCTAGAatggccagtctcgagaaggg	CCATGGATCCTCTAGAccatgcatcgaaaaagtccg	148	1175
CMS267C	TTCGTTGACCTCTAGAatgagcgatgtttcgctcca	CCATGGATCCTCTAGAccattgagagtttcgcgcac	86	1147
CMN333C	TTCGTTGACCTCTAGAatgggcgtcgctctttctct	CCATGGATCCTCTAGAcaaaagagaaccagcaactg	94	788
CME153C	TTCGTTGACCTCTAGAatgaagaggccgaggagacg	CCATGGATCCTCTAGAgcaggtgctaggactttggt	50	767
CMM188C	TTCGTTGACCTCTAGAatgggggatttgggtgaaca	CCATGGATCCTCTAGAgtttgcgaccaataagcgct	38	2719
CMC015C	TTCGTTGACCTCTAGAatggcgtacctcgtcttttt	CCATGGATCCTCTAGAgaactcttgcacttccgaca	155	351
CMS299C	TTCGTTGACCTCTAGAatggaacagtcgagcgcatt	CCATGGATCCTCTAGAcgcaatgagaactttgaaag	82	550
CMT073C	TTCGTTGACCTCTAGAatgaactcggcagcaatagc	CCATGGATCCTCTAGAtcgcacgagttcagcgcgag	45	917
CML055C	TTCGTTGACCTCTAGAatgcaaacgatcaactgcag	CCATGGATCCTCTAGAtctgtctttcggagggagga	146	1316
CMN243C	TTCGTTGACCTCTAGAatgccgtttcggagagtgct	CCATGGATCCTCTAGAaaagaccgcgtctgcgcgtg	74	672
CMM132C	TTCGTTGACCTCTAGAatgcgagacacctcctggcc	CCATGGATCCTCTAGAcgtggttacggagccgttta	60	604
CMT071C	TTCGTTGACCTCTAGAatgcggcctttcggttccgt	CCATGGATCCTCTAGAtgacgaagctagccgccgtg	33	590
CMC080C	TTCGTTGACCTCTAGAatgacgagcgacaagttcgc	CCATGGATCCTCTAGAaaacgcaaaagatcgtatca	114	365

CMM286C	TTCGTTGACCTCTAGAatgtttgcgggcgcacctgt	CCATGGATCCTCTAGAagttacgacaacacgctttc	80	490
CMD118C	TTCGTTGACCTCTAGAatggcttctccagatccgagtctcg	CCATGGATCCTCTAGAactcaaaatgagaaacagga	138	549
CMT420C	TTCGTTGACCTCTAGAatggaaaccccagtgggttt	CCATGGATCCTCTAGAcggacaaagcactacgacag	81	398
CMK172C	TTCGTTGACCTCTAGAatgaaactcggctaccttac	CCATGGATCCTCTAGAtgtgatgaaggcccaatcc	69	327
CMS393C	TTCGTTGACCTCTAGAatgctgctctttgcgaaccc	CCATGGATCCTCTAGAaccccgactgccaccagtga	88	321
CMI240C	TTCGTTGACCTCTAGAatgtttctctgttcaactgg	CCATGGATCCTCTAGAaatggcctcggcgtccagca	114	136
CMR006C	TTCGTTGACCTCTAGAatggcgtacggcaagacttg	CCATGGATCCTCTAGAaccgacgagtacgtacgcgg	65	303
CMT381C	TTCGTTGACCTCTAGAatggccttcgtttcctcttg	CCATGGATCCTCTAGAaccaccgccggtatccgaga	84	332
CMD146C	TTCGTTGACCTCTAGAatgagcctgtcgattcaatc	CCATGGATCCTCTAGAccaagagacctgcggcccca	97	311
CMJ263C	TTCGTTGACCTCTAGAatgctttcagtgaagcgctt	CCATGGATCCTCTAGActgcgggtgccgctgggccc	117	516
CMR113C	TTCGTTGACCTCTAGAatgctttctcggcctgtagg	CCATGGATCCTCTAGAacgaccatactcaacgtggt	95	230
CMH111C	TTCGTTGACCTCTAGAatgtttgttagtggttgcaa	CCATGGATCCTCTAGAccgtttccgcagcgcaaacc	100	240
CME186C	TTCGTTGACCTCTAGAatgtccttaaacaaggagtt	CCATGGATCCTCTAGAtttccgacaaccacgtagga	59	656
CMG147C	TTCGTTGACCTCTAGAatgtcgacggctaataactt	CCATGGATCCTCTAGAggcacgtgccacgtccacgc	53	641
CML197C	TTCGTTGACCTCTAGAatggttatgaaacattttcg	CCATGGATCCTCTAGAtctagtctcctcttgagtaa	145	775
CMO037C	TTCGTTGACCTCTAGAatgcacctgccagcgcatcc	CCATGGATCCTCTAGAttggttattgagtagcgcac	160	1092
CMT459C	TTCGTTGACCTCTAGAatgggctttgtctcgggacg	CCATGGATCCTCTAGAcgcgccggcgtctcggttcc	101	824
CMM045C -1stMet	TTCGTTGACCTCTAGAatgacagccaaggttgaatc	CCATGGATCCTCTAGAtccaatgagcggcgtgaaga	96	476
CMM045C -2ndMet	TTCGTTGACCTCTAGAatgagcgagaaggaactgaa	CCATGGATCCTCTAGAccacaagcgatgatacccgc	66	476
CMJ201C -1stMet	TTCGTTGACCTCTAGAatgattgatgctagagacgg	CCATGGATCCTCTAGAgcctttcagggcgaagacgt	78	412
CMJ201C -2ndMet	TTCGTTGACCTCTAGAatgcgtgcggtccgaaatcg	CCATGGATCCTCTAGAgcctttcagggcgaagacgt	34	412
CMK291C -1stMet	TTCGTTGACCTCTAGAatgaatacaagcatatatga	CCATGGATCCTCTAGAggccgcgtatgccgacgagc	119	499
CMK291C -2ndMet	TTCGTTGACCTCTAGAatgtgcgcgtcggaccaggc	CCATGGATCCTCTAGAgccaactttggccagcgacg	103	499
CMK291C -3rdMet	TTCGTTGACCTCTAGAatgattacgaatcgggagtt	CCATGGATCCTCTAGAgccaactttggccagcgacg	72	499
CMK115C	TTCGTTGACCTCTAGAatggaaaaacatttaccgat	CCATGGATCCTCTAGAacgctgtcgacagaaagtaa	87	773
CML080C	TTCGTTGACCTCTAGAatgcttcgggtatttagact	CCATGGATCCTCTAGAtgtcgcctggagcacagaca	58	450
CMT072C	TTCGTTGACCTCTAGAatgctttggcttggccgagc	CCATGGATCCTCTAGAcgcgacagtgctttgcggcg	151	632
CMK139C	TTCGTTGACCTCTAGAatgcgctctttcgtcacagg	CCATGGATCCTCTAGAcaaagccgctggtcgatgaa	68	300

CMT074C	TTCGTTGACCTCTAGAatgtcgagcttgtcattctg	CCATGGATCCTCTAGAtgctcgttccggccggttca	98	347
CMC137C	TTCGTTGACCTCTAGAatgcaggttcctaggattgt	CCATGGATCCTCTAGAttcggcacctcgaaggacgt	52	256
CMR380C	TTCGTTGACCTCTAGAatgacgggagagagtgtgag	CCATGGATCCTCTAGAatcgagtgctggctcaacgc	159	1145
CMA042C	TTCGTTGACCTCTAGAatggatgcacctgcgcctgc	CCATGGATCCTCTAGActcgagggcgcctcgtaccg	46	438
CME087C	TTCGTTGACCTCTAGAatgctgtgttctcgtggcac	CCATGGATCCTCTAGAaagcttgccaccgaaagagc	54	584
CMT151C	TTCGTTGACCTCTAGAatggctgctcgtggtacctc	CCATGGATCCTCTAGAtgctagcgtgtaggtgacgg	96	1016
CMP157C	TTCGTTGACCTCTAGAatgttcgtgcagagaacgtt	CCATGGATCCTCTAGAcgaacccgtaccagaggata	144	778
CMS254C	TTCGTTGACCTCTAGAatggtggcggtaggcaagtt	CCATGGATCCTCTAGAgctgccaattgttgccacat	271	1117
CMJ049C	TTCGTTGACCTCTAGAatgtccacaaaacgtgcagg	CCATGGATCCTCTAGAggagtacgaatctgcctgac	150	780
CMH156C	TTCGTTGACCTCTAGAatgccgtcggtggacaccag	CCATGGATCCTCTAGAacggaaggcttcgtcgattg	137	547
CMA056C	TTCGTTGACCTCTAGAatgtggtttgctcttgcgct	CCATGGATCCTCTAGAacctcgcagtcgcaacatgc	66	1035
CMR088C	TTCGTTGACCTCTAGAatgtatatagtctgcgtgca	CCATGGATCCTCTAGAgtttcccaaaccaggcagaa	59	292
CMF053C	TTCGTTGACCTCTAGAatggcgaacagggagagcag	CCATGGATCCTCTAGAaaagtccttcgcatccacgc	120	1089
CMT274C	TTCGTTGACCTCTAGAatgtcgtacgtggacccatt	CCATGGATCCTCTAGAtccgaatccatgcagtagaa	129	442
CMP032C	TTCGTTGACCTCTAGAatggatcggcgctatgatga	CCATGGATCCTCTAGAacccgtgagaccgcccgcgc	78	257
CMT308C	TTCGTTGACCTCTAGAatgtttgcaggctgtagcct	CCATGGATCCTCTAGAttcagcagcaattggacgct	99	765
CML191C	TTCGTTGACCTCTAGAatggaagatatgggaagttt	CCATGGATCCTCTAGAaacaagtactggtttctcag	75	433
CMO177C	TTCGTTGACCTCTAGAatgtgcagtgcgcgagttgg	CCATGGATCCTCTAGAatttcggagggagcgaatgc	93	562
CMR500C	TTCGTTGACCTCTAGAatggcgttcgttactttcgg	CCATGGATCCTCTAGAcggcacacatacacgcaccg	139	1284
CMT312C	TTCGTTGACCTCTAGAatgccgggcaacgattgcag	CCATGGATCCTCTAGAtatccggataccgtgcgggc	261	600
CMC146C	TTCGTTGACCTCTAGAatggatgctgaacaacgttc	CCATGGATCCTCTAGAgttcagttcgttaccgcgcc	61	741
CMS228C	TTCGTTGACCTCTAGAatgggaaagacagacttgga	CCATGGATCCTCTAGAgacataagcgcgaacatttc	97	384
CMP278C	TTCGTTGACCTCTAGAatgagcaaccttaaacaggt	CCATGGATCCTCTAGActgaacctccgagccaaagg	92	92
CMS372C	TCGTTGACCTCTAGAatgacgctcgttacagtagc	CATGGATCCTCTAGActtggcgtgcgcgttgttgg	129	129
CMT175C	TCGTTGACCTCTAGAatgactcgcctacaaccgct	CATGGATCCTCTAGAcccgaaacccatggccaacg	74	321
CMM126C	TCGTTGACCTCTAGAatgaacctgaatatgatcct	CATGGATCCTCTAGAgccgaggaacatcacccccg	106	289
CME109C	TTCGTTGACCTCTAGAatgtcgcaaacatgggaaaa	CCATGGATCCTCTAGAagagaccagagcttccaccc	122	436
CMP142C	TTCGTTGACCTCTAGAatgattcatggtaacgagtc	CCATGGATCCTCTAGAcaccgcttcgtgcaagcgat	208	446
CMR130C	TTCGTTGACCTCTAGAatggcggcgactagccctga	CCATGGATCCTCTAGAttccgctgctttcttgtcaa	83	371

CMB069C	TTCGTTGACCTCTAGAatggctttttgcgatttgag	CCATGGATCCTCTAGAtgcttgtgtcaatcgaaaca	188	1040
CMP267C	TCGTTGACCTCTAGAatgctaaaaaacatggagag	CATGGATCCTCTAGAaccgtccagggacaaccaac	180	944
CMH204C	TCGTTGACCTCTAGAatgggcgctttttgtgacca	CATGGATCCTCTAGAacacaaaaacacatgctcta	193	889
CMQ413C	TCGTTGACCTCTAGAatgggttgtacttgcgggga	CATGGATCCTCTAGAataacacgcatgggagcgaa	114	388
**HA tag construct**				
CMJ027C	CTGCAGTTAATTAATatgtgggtgtcttgtatttc	TGGGTAATTAATTAAttgcgcatcgtacgcaccca	452	452
CML329C	CTGCAGTTAATTAATatgtcgcacgaacttgtcca	TGGGTAATTAATTAAggtcgatacgccatccccct	477	477
**EGFP-*C*-terminal peptide construct**				
CMK115C	GACGAGCTGTACAAGgcccacattcacaaatacgg	CGGGCGGCCGCTTTAgagacgcgggaatcgccaac	67	773
CMR380C	GACGAGCTGTACAAGaatggcggtgcaattgcact	CGGGCGGCCGCTTTAaagtgctgctcgcaaccgtc	67	1145
CMA042C	GACGAGCTGTACAAGcgggagctgcctcggattcg	CGGGCGGCCGCTTTAagccagtcgccgaatgagaa	67	438

Supplementary Table 3. A summary of other enzymes related to acyl lipid metabolism in *C. merolae*. This table is a list of acyl lipid metabolic enzymes in *C. merolae.* Column 4 indicates the results of subcellular localization analysis in this study. Column 5 is a summary of the results of prediction of subcellular localization using three different programs. TargetP and the PredAlgo can predict plastidic, mitochondrial and secretory pathway proteins. Proteins predicted as targeted to other subcellular compartments were indicated "Other". WoLF PSORT can predict various subcellular localizations of proteins. Results of prediction using the WoLF PSORT indicated subcellular localization(s) having the highest score. Abbreviations: CM; cytoplasmic membrane, Cyt; cytosol, ER; endoplasmic reticulum, Ext; extracellular, Mt; mitochondrion, Nuc; nucleus, Per; peroxisome, PM; Plasma membrane, Pt; plastid, Pt-genome; genes encoded in the plastid genome, SP; secretary pathway, Ves; vesicle.

1. Enzyme name	2. Abbreviation of enzyme name and/or gene name	3. Locus tag	4. Subcellular localization	5. Result of prediction of subcellular localization		
				TargetP	WoLF PSORT	PredAlgo
**Lipase**						
Triacylglycerol lipase	TAGL	CMT151C	ER	Pt	Nuc, PM	Pt
		CMP157C	Pt	Mt	Pt	Pt
		CMS254C	ER	SP	PM	Other
		CMH156C	Cyt	Other	Cyt	Other
		CMA056C	ER	Mt	Pt	SP
		CMR088C	Pt	Mt	Mt	Pt
		CMJ049C	ER	Mt	ER	Other
		CMF053C	ER, CM	Mt	Nuc	Pt
Phospholipase A1 (PA-preferring phospholipase A1?)	PLA1	CMP267C	Cyt	Other	Nuc	Other
		CMH204C	Cyt	Other	PM	Other
		CMQ413C	Ves, CM	Other	Nuc	Pt
Phospholipase A2	PLA2	CMR500C	Nuc, Pt	Pt	Nuc	Pt
		CMT312C	ER, Pt	Other	Pt	Other
Phospholipase A2 activating protein	PLAP	CMC146C	Cyt	Other	Pt	Other
Phospholipase C	PLC	Not detected				
Phospholipase D	PLD	Not detected				
Hydrolase (Lipase?)	-	CMT274C	Cyt	Other	Cyt	Other
		CMP032C	Cyt, CM	Other	Ext	Other
		CMT308C	Pt	Mt	Pt	Mt
		CML191C	ER	Other	Pt	SP
		CMO177C	ER	SP	Pt	Pt
		CMS228C	Cyt	Other	Pt	Other
**Lipid trafficking**						
Phosphatidic acid transporter (TGD1-3 complex)	TGD1	CMV212C	Pt-genome			
	TGD2	CMV057C	Pt-genome			
	TGD3	CMR180C	ER	Mt	Pt	Mt
		CMH235C	ER	Other	PM	Other
		CMJ039C	ER	Mt	PM	SP
		CMR388C	Pt	Pt	Pt	Pt
Phosphatidic acid transporter (TGD4)	TGD4	Not detected				
Acyl-CoA binding protein	ACBP	CMP278C	Cyt	Other	Pt	Other
Flippase (P4 type-ATPase)	ALA1	CMR306C	ER, CM	Other	PM	Other

	ALA2	CMS375C	Cyt, Nuc	Mt	Cyt	Mt
ALA-interacting subunit	ALIS	CMT246C	ER, CM	Other	Nuc	Other
**Fatty acid activation**						
Acyl-CoA thioesterase	ACT	CMJ263C	Mt	Mt	Mt	Pt
		CMR113C	Mt	Mt	Pt	Pt
Long-chain acyl-CoA synthetase	LACS	CME186C	Cyt	Other	Cyt	Other
		CMG147C	Cyt	Other	Pt	Mt
		CML197C	Cyt, Pt	Other	Cyt	Other
		CMO037C	Cyt	Other	Pt	Other
		CMT459C	Pt	Pt	Pt	Pt
**PI signaling**						
Phosphatidylinositol-4-kinase	PI4K	CMI125C	ER	Other	Pt	Mt
		CMS267C	Cyt, CM	Other	Nuc	Other
Phosphatidylinositol-4-phosphate 5-kinase	PIP5K	CMN333C	ER	Other	Nuc	Other
		CME153C	Cyt, CM	Mt	Pt, Nuc	Pt
**Biotin-dependent carboxylase**						
Holocarboxylase synthetase	HCS	CMC080C	Pt, Cyt	Mt	Pt	SP
Methylcrotonyl-CoA carboxylase	MCC (MCCA)	CMT073C	Mt	Pt	Cyt	Other
	(MCCB)	CMT071C	Mt	Mt	Pt	SP
Propionyl-CoA carboxylase	PCC (PCCA)	CMN243C	Mt	Mt	Mt	Other
	(PCCB)	CMM132C	Mt	Mt	Pt, Pt_Mt	Pt
Carbamoylphosphate synthase (Multifunctional type)	CAR (CAR1)	CMQ255C	Not analyzed	Other	Cyt	Other
Carbamoylphosphate synthase (Multisubunit type)	CAR (CarA)	CMV036C	Pt-genome			
	(CarB)	CML055C	Pt	Pt	Cyt	Pt
Biotin carboxyl carrier protein	BCCP	CMC015C	Pt	Mt	Pt	Pt
